# Investigating the Pharmacological Basis of Xiaochaihu-Tang in Diabetic Cognitive Impairment: A Network Pharmacology Approach

**DOI:** 10.3390/brainsci16101005

**Published:** 2026-09-23

**Authors:** Bobeen Joo, Dong-Uk Kim, Bitna Kweon, Gi-Sang Bae

**Affiliations:** 1 Department of Pharmacology, School of Korean Medicine, Wonkwang University, Iksan 54538, Republic of Korea; hibobeen@daum.net (B.J.);; 2 Hanbang Cardio-Renal Syndrome Research Center, School of Korean Medicine, Wonkwang University, Iksan 54538, Republic of Korea; 3 Research Center of Traditional Korean Medicine, Wonkwang University, Iksan 54538, Republic of Korea

**Keywords:** diabetic cognitive impairment, Xiaochaihu-tang, network pharmacology

## Abstract

**Highlights:**

**What are the main findings?**

- Integrated network pharmacology and transcriptomic dataset analysis predicted TLR4, STAT3, and BCL2 as core therapeutic targets of Xiaochaihu-Tang against diabetic cognitive impairment.
- Molecular docking analysis predicted that key compounds, including stigmasterol and isorhamnetin, bind to primary target proteins, thereby blocking inflammation, apoptosis, and metabolic dysfunction.

**What are the implications of the main findings?**

- Demonstrated the multi-component, multi-target rationale of Xiaochaihu-Tang for simultaneously regulating complex pathological processes in diabetic cognitive impairment.
- Established foundational hypotheses based on precise binding site predictions to enhance the efficiency of future in vitro and in vivo experimental validations of Xiaochaihu-Tang.

**Abstract:**

**Background/Objectives:** Diabetic Cognitive Impairment (DCI) involves cognitive impairment resulting from diabetes-triggered metabolic abnormalities and neuroinflammation; however, the therapeutic mechanisms of Xiaochaihu-tang (XCHT) remain unclear. **Methods:** This study systematically explored the potential pharmacological mechanisms of XCHT on DCI using network pharmacology and molecular docking analysis. **Results:** Based on 263 bioactive compounds from XCHT, 1017 intersecting genes with DCI were identified. Protein–protein interaction, GO, and KEGG analyses narrowed these to 22 major target genes involved in inflammation, apoptosis, and metabolic pathways. Validation using public transcriptomic datasets (GSE161355, GSE122063, GSE5281) and ROC–AUC analysis identified TLR4, BCL2, and STAT3 as top candidate targets. Furthermore, molecular docking analysis showed a notably stable binding interaction for TLR4–Stigmasterol, suggesting a possible structural basis by which XCHT may modulate TLR4 signaling in DCI. **Conclusions**: These findings suggest that XCHT may multi-dimensionally regulate DCI, providing essential foundational data for future cellular and animal experimental validation.

## 1. Introduction

Diabetes mellitus (DM), a metabolic disorder characterized by chronic hyperglycemia, has become a serious public health concern due to its continuously rising global prevalence [1,2]. Type 2 diabetes mellitus (T2DM) accounts for 85–95% of all diabetes cases, driven primarily by peripheral insulin resistance (IR) and pancreatic β-cell dysfunction as its core pathophysiological mechanisms. This intractable chronic condition sustains persistent hyperglycemia, which precipitates a broad spectrum of systemic complications, including nephropathy, retinopathy, cardiovascular diseases, and peripheral neuropathy [3,4,5]. Recently, beyond these traditional complications, renewed attention has been paid to the association between T2DM and cognitive decline, which is characterized by deficits in various cognitive domains, including attention, memory, and executive function [1,6]. This condition, known as Diabetic Cognitive Impairment (DCI), is closely linked to complex pathophysiological mechanisms such as insulin resistance, inflammation, dyslipidemia, oxidative stress, mitochondrial dysfunction, and dysregulated autophagy [7,8]. Particularly, as the rising prevalence of Type 2 diabetes coincides with the global trend of increased life expectancy, there are growing concerns that DCI will place a massive burden on future healthcare systems [9]. Therefore, it is urgent to clearly understand the complex pathophysiology of DCI and to identify molecular targets and signaling pathways that can lead to the development of innovative therapies.

In Traditional Korean Medicine (TKM), diabetes falls under the category of ‘Xiaoke’, which signifies severe thirst. Traditionally, its etiology is attributed to Yin deficiency with internal heat, Spleen and Stomach weakness, and Kidney Yin deficiency [10,11]. These pathological changes lead to fluid depletion and metabolic imbalances, and if prolonged, this condition fails to adequately nourish the brain, resulting in neurological symptoms such as cognitive decline, amnesia, and dementia [12]. To fundamentally restore the intricately intertwined imbalances of the central nervous system and systemic metabolism, a holistic treatment strategy that simultaneously regulates multiple pathways is required, moving beyond a single-component approach [13,14]. Accordingly, active interventions using TKM are being implemented in clinical practice to control the exacerbation of diabetes and its neurological complications [15].

Xiaochaihu-tang (XCHT; also known as Sosiho-tang or Soshiho-tang in Korea, and Sho-saiko-to in Japan) is a traditional prescription recorded in the classical medical treatise, Shanghan Lun [16]. Composed of seven medicinal herbs—Bupleuri Radix (BR), Scutellariae Radix (SR), Ginseng Radix (GR), Glycyrrhizae Radix et Rhizoma (GRR), Zingiberis Rhizoma Recens (ZRR), Zizyphi Fructus (ZF), and Pinelliae Tuber (PT)—it is widely used for various conditions [17,18,19]. Notably, the modern “Clinical Practice Guidelines for Type 2 Diabetes in Korean Medicine” recommends considering treatments such as herbal medicine, acupuncture, and moxibustion for patients with prediabetes and type 2 diabetes. Specifically, it recommends using XCHT alone or in combination with oral hypoglycemic agents when symptoms such as dry mouth, dry throat, and chest tightness are present [20]. This is believed to be related to the mechanism by which XCHT alleviates internal heat and inflammatory responses. Beyond clinical guidelines, numerous recent studies have scientifically validated the efficacy of XCHT. For example, XCHT has been reported not only to alleviate memory decline in an amyloid-β (Aβ)-induced cognitive impairment model but also to significantly improve blood glucose control in clinical settings when co-administered with insulin in diabetic patients [21,22]. This suggests that XCHT may be effective for both central nervous system protection and systemic metabolic regulation. Nevertheless, the direct effects and molecular mechanisms of XCHT on diabetic cognitive impairment (DCI), a condition where these two pathologies intersect, remain to be clearly elucidated.

To overcome the limitations of mechanism elucidation, network pharmacology has recently emerged as a pivotal analytical tool for interpreting the pathophysiology of complex diseases [23]. This approach integrates systems biology, bioinformatics, and pharmacology to comprehensively analyze interactions among bioactive compounds, target genes, and biological pathways. By doing so, it enables the exploration and prediction of potential therapeutic targets, thereby allowing for a holistic interpretation of disease-regulatory mechanisms [24]. Given the characteristics of TKM formulations, in which multiple bioactive compounds act to regulate complex targets, this methodology is the optimal approach for interpreting the ‘multi-component, multi-target’ interactions of these formulations [25]. Indeed, recent studies suggest that multi-target modulation of diabetic hyperglycemia and its subsequent complications is crucial for disease management [26,27,28]. This aligns closely with the pathophysiological characteristics of DCI, wherein complex pathologies such as inflammation, oxidative stress, and neurovascular dysfunction are intricately intertwined [29,30]. Although network pharmacology has been widely utilized to investigate XCHT in conditions such as colorectal cancer, liver fibrosis, postmenopausal osteoporosis, and tinnitus, the system-level prediction of its potential target genes and biological pathways in DCI remains unexplored [31,32,33,34].

Therefore, this study aimed to comprehensively analyze the complex interaction network between XCHT and DCI using a network pharmacology approach. Ultimately, the goal was to identify key targets and biological pathways involved in DCI regulation, thereby providing a pharmacological basis to support the clinical application of XCHT.

## 2. Results

### 2.1. Active Components and Common Genes of the Herbs in XCHT

The herbs comprising XCHT were identified as seven types: BR, SR, GR, GRR, ZRR, ZF, and PT. The main compounds of each herb were compiled by collecting PubChem CIDs from the PubChem database based on physicochemical information provided in OASIS. As a result, a total of 359 compounds were identified. The number of compounds per herb was as follows: BR (8), SR (12), PT (93), GR (16), GRR (154), ZRR (26), and ZF (50).

To screen bioactive compounds, criteria for oral bioavailability assessment (Lipinski violation ≤ 1, bioavailability > 0.1, total polar surface area (TPSA) < 140 Å^2^) were applied. A total of 263 compounds met these conditions, distributed as follows: SR (6), PT (89), GR (2), GRR (99), ZRR (26), and ZF (46). No bioactive compounds were identified in BR (Table S1).

For BR and GR, since no bioactive compounds were identified or only a very small number were identified, additional compound screening was performed using the TCMSP database. Among the retrieved compounds, those with available PubChem CID, oral bioavailability ≥ 30%, and drug likeness ≥ 0.18 (15 compounds from BR and 9 from GR) were included in the analysis (Table S2).

For target gene analysis of bioactive compounds, only compounds with available Chemical-Gene Co-occurrence information were considered. After excluding duplicates and non-gene entries, a total of 1254 target genes were selected. Using the GeneCards database, a total of 9940 genes associated with DCI were retrieved. Comparison with the target genes of the bioactive compounds in XCHT identified 1017 overlapping genes, corresponding to approximately 81.1% of all XCHT target genes (Figure 1 and Table S3).

### 2.2. Hub Gene Identification via PPI Network Analysis of XCHT in DCI

Based on cross-gene interactions, a protein–protein interaction (PPI) network was constructed. The initial network comprised a total of 1017 nodes and 4368 edges. Subsequently, major target genes were selected stepwise using network centrality metrics. In the first step, filtering based on the average values of degree centrality (DC), betweenness centrality (BC), and closeness centrality (CC) (DC > 0.008858, BC > 0.006259, CC > 0.242063) resulted in a reduced network consisting of 123 nodes and 683 edges. In the second step, additional screening using the same criteria resulted in 22 nodes and 99 edges, with threshold values of DC > 0.098361, BC > 0.016162, CC > 0.391026. The final network comprised 22 nodes and 99 edges (Figure 2 and Table 1).

### 2.3. Functional Enrichment Analysis of Major Target Genes

#### 2.3.1. Functional Analysis of Major Target Genes

Gene Ontology (GO) functional enrichment analysis of the 22 major target genes identified 1179 Biological Process (BP) terms, with enrichment observed in processes related to cell death, gene regulation, and signaling pathways. Representative items included ‘Regulation of miRNA Transcription (GO:1902893)’, ‘Regulation of Apoptotic Process (GO:0042981)’, ‘Regulation of Inflammatory Response (GO:0050727)’, ‘Regulation of Nitric Oxide Biosynthetic Process (GO:0045428)’, and ‘Epidermal Growth Factor Receptor Signaling Pathway (GO:0007173)’. Analysis of Cellular Component (CC) terms identified 65 items, with ‘Membrane Raft (GO:0045121)’, ‘Nucleus (GO:0005634)’, ‘Caveola (GO:0005901)’, and ‘Early Endosome (GO:0005769)’ among the enriched components, indicating cellular structures associated with signal transduction and transcriptional regulation. Molecular Function (MF) analysis yielded 125 items, including ‘DNA-binding Transcription Factor Binding (GO:0140297)’, ‘RNA Polymerase II-specific DNA-binding Transcription Factor Binding (GO:0061629)’, ‘Ubiquitin Protein Ligase Binding (GO:0031625)’, and ‘MAP Kinase Activity (GO:0004707)’, suggesting involvement in gene expression regulation, transcriptional control, protein processing, and signal transduction (Figure 3 and Tables S4–S6).

#### 2.3.2. Pathway Analysis of Major Target Genes

Kyoto Encyclopedia of Genes and Genomes (KEGG) pathway enrichment analysis identified a total of 197 pathways. Among the top 30 pathways ranked by adjusted *p*-value, those closely associated with the pathophysiology of DCI included ‘NOD-like receptor signaling pathway’, ‘Toll-like receptor signaling pathway’, ‘Apoptosis’, ‘Lipid and atherosclerosis’, ‘AGE-RAGE signaling pathway in diabetic complications’, ‘HIF-1 signaling pathway’, ‘FoxO signaling pathway’, ‘Fluid shear stress and atherosclerosis’, ‘PI3K-Akt signaling pathway’, ‘MAPK signaling pathway’, and ‘IL-17 signaling pathway’. These pathways were primarily related to inflammatory responses, oxidative stress, programmed cell death, vascular dysfunction, and metabolic dysregulation, and were therefore selected as core pathways for subsequent analysis (Figure 4 and Table S7).

### 2.4. Construction of the Prescription-Herb-Compound-Target-Pathway (PHCTP) Network Related to XCHT and DCI

Analysis of items closely associated with DCI in the PHCTP network of XCHT, listed in descending order of DC score, identified the top eight bioactive compounds: Formononetin, Wogonin, Catechin, Epicatechin, Stigmasterol, Kaempferol, Isorhamnetin, Quercetin. Among the major target genes, the top 10 items were IL6, INS, TNF, AKT1, BCL2, TP53, PPARG, TLR4, EGFR, STAT3; however, ALB and ESR1 were not retained in the PHCTP network. The 11 pathways examined included ‘Lipid and atherosclerosis’, ‘PI3K-Akt signaling pathway’, ‘MAPK signaling pathway’, ‘Fluid shear stress and atherosclerosis’, ‘FoxO signaling pathway’, ‘HIF-1 signaling pathway’, ‘AGE-RAGE signaling pathway in diabetic complications’, ‘NOD-like receptor signaling pathway’, ‘Apoptosis’, ‘Toll-like receptor signaling pathway’, and ‘IL-17 signaling pathway’ (Figure 5).

### 2.5. Identification of Core Target Genes via Transcriptomic Validation

The 22 genes selected as major target genes were further evaluated using transcriptomic datasets related to DCI and Alzheimer’s disease (AD) (GSE161355, GSE122063, and GSE5281), through which AKT1, ALB, BCL2, CAV1, CTNNB1, FOXO1, INS, IL6, JUN, MAPK3, PPARG, STAT3, and TLR4 were identified as core target genes.

These thirteen genes each exhibited log_2_ fold-change values with identical signs, indicating consistent expression trends (up- or down-regulation) (Figure 6 and Table 2). As a consistent direction of expression was confirmed across all 13 genes, the *p*-values for each gene were integrated using Fisher’s method to derive a combined *p*-value. The resulting combined *p*-values were adjusted for multiple comparisons across the 13 genes using the Benjamini–Hochberg (BH) procedure. Based on a false discovery rate (FDR) threshold of <0.05, a total of 11 genes (ALB, TLR4, BCL2, STAT3, CAV1, PPARG, FOXO1, CTNNB1, JUN, INS, and MAPK3) reached statistical significance, whereas AKT1 and IL6 failed to meet this criterion and were excluded from subsequent analyses (Table 3).

### 2.6. Identifying Core Target Gene Pathways and Assigning Importance

To rank the candidate targets for subsequent analysis, Receiver Operating Characteristic (ROC) area under the curve (AUC) values were evaluated and averaged across the three GEO datasets. Consequently, the top three genes with the highest mean AUC values (BCL2, STAT3, and TLR4) were prioritized for downstream molecular docking (Figure 7 and Table 4).

### 2.7. Construction of the miRNA–mRNA Regulatory Network

Using experimental and prediction-based databases, miRNA interactions were identified, and weights were assigned to the primary core target genes and corresponding miRNA sets. The highest composite score reached 9 points (classified as High), and a total of 21 miRNA–gene interaction sets were identified. The BCL2- and STAT3-related miRNA sets each comprised 20 interaction pairs, with identical scores of 4 points in miRTarBase, 2 points in TargetScan, and 3 points in ENCORI. The miR-7-5p–TLR4 pair achieved a score of 6 points in miRTarBase and 3 points in ENCORI but showed a low TargetScan prediction score of 0 points.

The primary target genes of the miRNAs were categorized as follows. The miR-15/16 family (miR-15a/b-5p, miR-16-5p, miR-195-5p, miR-503-5p), miR-181 family (miR-181a/b/c/d-5p), and miR-34/449 family (miR-34a/c-5p, miR-449a) were all confirmed to share BCL2 as a common target. The miR-17/92 cluster (miR-17-5p, miR-20a/b-5p, miR-93-5p, miR-106a/b-5p) and the miR-125 family (miR-125b-5p), C19MC cluster (miR-519d-3p) were primarily associated with STAT3. Only the miR-7 family (miR-7-5p) was identified as interacting with TLR4 (Figure 8 and Table 5).

### 2.8. Molecular Docking of BCL2, STAT3, and TLR4 with Candidate Bioactive Compounds

Molecular docking was conducted on BCL2, STAT3, and TLR4 proteins for the top eight bioactive compounds based on the DC criteria of the PHCTP network, yielding 24 binding models with Vina scores ranging from −10.4 to −5.5 kcal/mol (Figure 9 and Table 6). For STAT3 and BCL2, docking scores among the eight candidate compounds were within a narrow range (approximately 1 kcal/mol), indicating broadly comparable predicted affinities rather than a clearly preferential ligand. In contrast, TLR4 displayed the most robust binding affinity among all docking pairs evaluated in the blind docking analysis; the TLR4–stigmasterol complex achieved the lowest Vina score of −10.4 kcal/mol, reflecting a markedly more stable binding conformation than the other candidates (Table 7). Given this substantial difference, the TLR4–stigmasterol interaction was selected for further evaluation. To verify the precise binding mode within the activation site, a structural superposition was performed using the reference TLR4–MD-2 complex (PDB ID: 3FXI). This comparison showed that stigmasterol occupied the deep hydrophobic cavity of MD-2, overlapping with the binding site of the reference ligand, LPS (Figure 10), and that stigmasterol stabilized its docking posture predominantly through hydrophobic interactions, consistent with the reference complex’s binding mode.

## 3. Discussion

Network pharmacology, a powerful analytical tool based on pharmacology, chemoinformatics, and bioinformatics, is utilized to study the complex, multi-target mechanisms of TKM [35]. This approach is highly effective in exploring integrated therapeutic mechanisms—which have been difficult to elucidate using conventional single-target approaches—given the complex pathological cascades characteristic of DCI. Our findings suggest that XCHT may potentially alleviate DCI symptoms through multifaceted network regulation, simultaneously modulating multiple pathways and targets rather than acting on a single molecular mechanism. Similar to prior network pharmacology studies that demonstrated XCHT’s broad, multi-target therapeutic potential in various disease models, our findings reflect a comparable multifaceted regulatory capacity [31,32,33,34]. Therefore, XCHT represents a promising candidate strategy that holds potential to address the complex pathological processes associated with DCI at multiple levels.

In pursuit of the specific network structures underpinning these multifaceted effects, we systematically screened the bioactive compounds of XCHT and cross-analyzed their potential targets with DCI-related genes. Through topological analysis of the integrated PPI network, we successfully narrowed down the vast target pool into key hub genes that are predicted to constitute key nodes in the pharmacological network of XCHT. Subsequent GO and KEGG enrichment analyses confirmed that these hub genes are deeply involved in critical pathological cascades, particularly those regulating inflammatory responses, vascular integrity, cell survival, and apoptosis. However, to prevent the complete loss of active constituents from BR and GR, the major herb of XCHT, SwissADME criteria were supplemented with TCMSP parameters for select herbs. This non-uniform screening across herbs represents a methodological limitation that may introduce selection bias. Additionally, target identification relied on literature-derived annotations from PubChem. Although this strategy minimizes false-positive predictions, it inherently carries an annotation-density bias, whereby less-studied phytochemicals may be excluded due to limited literature coverage rather than a genuine absence of pharmacological activity.

To further explore the potential multi-target mechanisms of XCHT against DCI, we categorized the 11 highly relevant KEGG pathways into three distinct functional modules: Vascular/Metabolic, Immune/Inflammatory, and Cellular Stress Response and Cell Survival. First, Vascular/Metabolic-related pathways, including ‘AGE-RAGE signaling pathway in diabetic complications’, ‘Lipid and atherosclerosis’, and ‘Fluid shear stress and atherosclerosis’, are activated under hyperglycemic conditions, driving the accumulation of oxidative stress and vascular dysfunction that ultimately contribute to the pathogenesis of DCI. Hyperglycemia-induced accumulation of advanced glycation end-products (AGEs) activates the receptor for AGE (RAGE), triggering reactive oxygen species (ROS) production and neuroinflammation [8]. This subsequently accelerates Aβ deposition and tau hyperphosphorylation in the brain, driving cognitive decline [36]. Additionally, linked to the ‘Lipid and atherosclerosis’ pathway, diabetic dyslipidemia exacerbates cognitive impairment by promoting cerebral small vessel atherosclerosis and white matter lesions, which reduce cerebral blood flow and inflict ischemic neuronal damage [37]. Finally, associated with the ‘Fluid shear stress and atherosclerosis’ pathway, altered shear stress in the diabetic microenvironment induces vascular endothelial dysfunction and increases BBB permeability [38,39].

Crucially, the disruption of vascular integrity and the resulting metabolic disorders act as primary triggers for central immune activation. The resulting infiltration of systemic noxious factors, coupled with local metabolic strain, disrupts glial homeostasis [40]. These immunometabolic changes induce the persistent activation of key immune/inflammation-related pathways, including the ‘Toll-like receptor signaling pathway’, ‘the NOD-like receptor signaling pathway’, and ‘IL-17 signaling pathway’, thereby perpetuating chronic neuroinflammation in diabetic conditions. Damage-associated molecular patterns (DAMPs) derived from hyperglycemia bind to TLRs and NLRs, triggering NLRP3 inflammasome formation and the release of proinflammatory cytokines such as IL-1β and TNF-α, which amplify the neuroinflammatory cascade in the brain [41,42]. IL-17 signaling further amplifies these responses by compromising the integrity of the BBB and facilitating the influx of peripheral immune cells into the central nervous system, ultimately worsening neuronal damage and cognitive decline [43].

Ultimately, this persistent neuroinflammatory cascade, combined with sustained metabolic stress, compromises intracellular machinery and drives neurons toward progressive degeneration and cell death [44]. Prolonged exposure to this cytotoxic microenvironment overactivates cellular stress kinases while progressively suppressing vital homeostatic survival networks, inevitably tipping the molecular balance toward structural neurodegeneration [45]. Consequently, ‘Cellular Stress Response and Cell Survival’-related pathways, including the ‘PI3K-Akt signaling pathway’, ‘MAPK signaling pathway’, ‘FoxO signaling pathway’, ‘HIF-1 signaling pathway’, and ‘Apoptosis’, are disrupted under chronic hyperglycemic conditions. Persistent hyperglycemia suppresses PI3K-Akt signaling and reduces Akt phosphorylation, weakening neuronal survival signals and ultimately disrupting the balance between pro- and anti-apoptotic proteins, leading to neuronal death [46]. In addition, MAPK signaling is overactivated by hyperglycemia and endoplasmic reticulum stress, promoting the production of inflammatory cytokines and exacerbating neuroinflammation [47]. The FoxO signaling pathway is abnormally activated in the insulin-resistant brain, driving autophagy and apoptosis due to oxidative stress [48]. Moreover, persistent activation of the HIF-1 signaling pathway shifts toward promoting apoptosis [49]. This complex disruption of cellular stress and survival pathways can lead to cognitive decline through widespread neuronal cell death.

To translate these pathological pathways into specific molecular interventions, we sought to evaluate the clinical significance of our findings. Given the relative scarcity of dedicated human DCI transcriptomic datasets in public repositories, and considering the substantial pathophysiological overlap between DCI and AD—particularly shared mechanisms such as brain insulin resistance, neuroinflammation, and cell death (often conceptualized under “Type 3 diabetes”)—we evaluated the 22 hub genes using transcriptomic datasets encompassing both DCI and AD patients (GSE161355, GSE122063, and GSE5281) [50,51]. Although AD and DCI remain distinct clinical entities, this integrative approach enabled us to capture robust, cross-disease target genes involved in cognitive impairment under metabolic stress. Through this cross-referencing, 11 genes were selected as candidate genes. Subsequently, mean AUC values across the three GEO datasets were used to rank the candidate genes, and TLR4, BCL2, and STAT3 were selected as the top three candidates for further investigation. In addition, considering that candidate genes are subject to post-transcriptional regulation, we evaluated their clinical relevance in DCI by cross-referencing them with miRNA networks. In the contexts of diabetes and AD, the BCL2, STAT3, and TLR4 axes are known to be severely dysregulated by pathological miRNA families (e.g., miR-15/16, miR-17/92, and miR-7), leading to accelerated apoptosis, chronic neuroinflammation, and metabolic burden [52,53,54]. These findings suggest that TLR4, BCL2, and STAT3 may play an important role in DCI pathogenesis. Building on this rationale, we subsequently performed molecular docking analysis to predict whether the active components of XCHT could bind to these key targets and potentially modulate their activity. However, given that DCI and AD are etiologically distinct entities, the possibility that a subset of the selected candidate genes reflects AD-specific pathology rather than diabetes-induced mechanisms cannot be entirely ruled out. Therefore, future studies utilizing dedicated DCI models are necessary to validate the disease specificity of these candidate genes.

Among the docking pairs evaluated, TLR4–Stigmasterol yielded the lowest Vina score (−10.4 kcal/mol), a value substantially lower than those observed for the other candidate pairs. For STAT3 and BCL2, Vina scores across the eight candidates were within a narrow range (approximately 1 kcal/mol; STAT3: −7.1 to −8.1 kcal/mol, BCL2: −5.6 to −6.5 kcal/mol), precluding confident identification of a preferentially binding ligand from docking scores alone. Given this pronounced difference, the TLR4–Stigmasterol interaction was selected for further structural evaluation.

The TLR4–MD-2 complex was employed as the structural template for this evaluation, because the hydrophobic pocket within the co-receptor MD-2 is essential for ligand recognition and the initiation of subsequent signaling [55,56]. Structural overlap analysis with the reference TLR4–MD-2 complex (PDB ID: 3FXI) showed that stigmasterol is stably positioned within this hydrophobic pocket, overlapping with the canonical ligand-binding interface. This specific site serves as the crucial region where diabetes-induced endogenous ligands bind to trigger the conformational shifts and subsequent dimerization of the TLR4 complex [57]. Therefore, these findings suggest that by potentially interfering with the docking of such endogenous inflammatory ligands, stigmasterol may help disrupt the assembly and activation of the TLR4 receptor complex required to initiate downstream signaling. This structural inference is consistent with a prior experimental report showing that stigmasterol suppresses the TLR4/NF-κB axis and attenuates microglial activation and neuroinflammation, suggesting a plausible mechanistic basis for the docking result observed in this study [58]. Nevertheless, as this remains merely a structural hypothesis derived from computer modeling, functional validation will be required to confirm the mechanism in the context of DCI.

This study successfully predicted the therapeutic mechanism of XCHT for DCI using open databases, clinical transcriptome data, miRNA regulatory networks, and molecular docking. The analysis suggested that XCHT may potentially attenuate DCI through the predicted regulation of multiple targets—such as TLR4, BCL2, and STAT3, which are involved in neuroinflammation, apoptosis, and metabolic regulation—rather than targeting a single target. These multifaceted in silico analysis results provide a scientific basis for potential anti-neuroinflammatory, neuroprotective, and metabolic regulatory effects of XCHT and will contribute to establishing future clinical treatment strategies for DCI and developing new multi-target therapies.

## 4. Materials and Methods

### 4.1. Identification of Active Compounds in XCHT Herbal Formula

In this study, the major compounds of each herbal component comprising XCHT were first investigated using the TKM portal OASIS (https://oasis.kiom.re.kr/; accessed on 1 October 2025). During this process, the physicochemical properties of each herb were reviewed, and the names and PubChem CIDs of the compounds were collected. Subsequently, to screen for potential bioactive compounds within XCHT, the PubChem CIDs and canonical SMILES strings for the identified compounds were extracted from the PubChem database (https://pubchem.ncbi.nlm.nih.gov/; accessed on 6 December 2025). The collected SMILES data were input into SwissADME (https://www.swissadme.ch/; accessed on 6 December 2025) to analyze their physicochemical and pharmacokinetic properties and to assess their oral bioavailability [59]. Active compounds were selected based on the following criteria: ≤1 violation of Lipinski’s Rule of Five, bioavailability score > 0.1, and TPSA < 140 Å^2^ [60]. For herbs where few or no bioactive compounds met these criteria, the TCMSP database (https://www.tcmsp-e.com/tcmsp.php; accessed on 6 December 2025) was additionally utilized. From this database, compounds with identifiable PubChem CIDs, oral bioavailability (OB) ≥ 30%, and drug-likeness (DL) > 0.18 were selected [61,62].

### 4.2. Screening of Intersecting Target Genes Related to the Active Compounds of XCHT and DCI

Target genes for the bioactive compounds of XCHT were collected using the “Chemical–Gene Co-Occurrences in the Literature” section of the PubChem database (https://pubchem.ncbi.nlm.nih.gov/; accessed on 6 December 2025). For the initial screening of collected genes, the Comparative Toxicogenomics Database (CTD; https://ctdbase.org/; accessed on 6 December 2025) was consulted; however, compounds with a PubChem CID exceeding 10,000 were mostly excluded from analysis due to the lack of linkage information with CTD, and a validation process was performed based on the NCBI Entrez Gene database (https://www.ncbi.nlm.nih.gov/gene; accessed on 6 December 2025) [63,64].

First, non-gene code data (e.g., EC number: EC 1.1.1.1, GO term: GO:0008150, UniProtID: P04637) were removed, and gene-like patterns were selected using regular expressions [65]. The selection criteria were set as strings consisting of 2 to 10 characters composed of uppercase English letters (A–Z) and numbers (0–9). Subsequently, only entries registered for Homo sapiens were retained via the Entrez Gene API to reduce redundancy, ultimately constructing a valid gene dataset.

Genes associated with DCI were identified using the GeneCards database (ver. 5.25, https://www.genecards.org/; accessed on 1 October 2025) using the query “[all] (“Diabetes”) AND [all] (“Cognitive disorder” OR “Cognitive decline” OR “Cognitive impairment” OR “Cognitive dysfunction” OR “Cognitive deficits” OR “Dementia”)” [66].

The intersecting target genes were derived by identifying overlapping items between the target genes of the collected bioactive compounds of XCHT and genes related to DCI, followed by duplicate removal.

### 4.3. Construction of the XCHT and DCI Network and Selection of Major Target Genes

The STRING database (https://string-db.org/; accessed on 15 December 2025) was utilized to analyze PPI between XCHT’s bioactive compounds and genes associated with DCI. The analysis was performed using Homo sapiens as the reference species. Only interactions with a combination score of 0.9 or higher were collected to construct the interaction network [13]. The constructed PPI network was visualized using the Cytoscape software (ver. 3.10.4; Cytoscape Consortium, San Diego, CA, USA; Java 17.0.12, Oracle Corporation, Austin, TX, USA), and its topological characteristics were analyzed. Centrality analysis metrics included DC, BC, and CC. Genes exhibiting values above the average for each metric were selected. This procedure was repeated once, and genes that consistently exhibited above-average values across all centrality metrics (DC, BC, and CC) were designated as hub genes, resulting in the identification of 22 major target genes [67,68].

### 4.4. Functional Enrichment Analysis

Functional Enrichment analysis was performed to characterize the biological functions and related signaling pathways of the major target genes. The analysis utilized Enrichr (https://maayanlab.cloud/Enrichr/; accessed on 10 January 2026) and was based on the GO and KEGG databases. The GO analysis classified results into three categories: BP, CC, and MF to summarize the functional characteristics of each item. Statistical significance was assessed based on the adjusted *p*-value. KEGG pathway analysis identified major metabolic and signaling pathways involving major target genes, with pathway significance also determined based on adjusted *p*-values. From the top 30 pathways identified by KEGG analysis, core pathways relevant to the pathophysiology of DCI were selected.

### 4.5. Construction of PHCTP Network Related to XCHT and DCI

To analyze the interaction between XCHT and DCI at multiple levels, a PHCTP network was constructed in four steps using Cytoscape (ver. 3.10.4, Java 17.0.12).

### 4.6. Selection of Core Target Genes

Based on the 22 genes identified as major target genes, candidate target genes were selected using public transcriptomic datasets related to DCI and AD [69]. Given the limited availability of transcriptomic datasets specific to diabetic cognitive impairment, AD-related datasets were additionally incorporated into the analysis, based on shared pathological mechanisms between the two conditions, including chronic neuroinflammation, neuronal apoptosis, and brain insulin resistance [70]. In this validation step, reproducibility was assessed primarily using genes that simultaneously satisfied consistent expression directionality and statistical significance in independent external datasets (GSE161355, GSE122063, GSE5281; Table 8) [71,72,73]. The selection criteria were as follows. First, to assess expression direction consistency, the sign (+/−) of the log_2_ fold change for each hub gene was compared across the three GEO series datasets. Genes showing a common expression trend across all datasets were considered reproducible. Second, for statistical significance, genes with FDR below 0.05 in at least two of the three datasets were finally selected as core target genes. Through this filtering process, a total of six core target genes were selected.

### 4.7. Identification and Validation of Primary Core Targets Using Network Centrality and ROC Curves

Based on KEGG analysis results for the major target genes (22 genes), we extracted signaling pathways associated with the initially selected core target genes (13 genes). Subsequently, a gene–pathway network was constructed using Cytoscape, and centrality metrics were analyzed to assess the connectivity of each node. Centrality analysis was performed based on DC, which reflects the relative importance of each gene according to its degree value. Furthermore, to assess the predictive performance of the core target genes, ROC analysis was performed using three GEO series datasets [74]. ROC analysis was conducted in Python (ver. 3.13.7; Python Software Foundation, Wilmington, DE, USA; using scikit-learn, NumPy [2.3.3], pandas [2.3.3], and Matplotlib [3.10.6]). Multi-probe expression values were summarized by arithmetic mean per gene. Across three GEO datasets (GSE161355, GSE122063, and GSE5281), disease cases were designated as the positive class (case = 1, control = 0). To maintain a uniform monotonic scoring direction, sign inversion was applied to the four down-regulated genes. Non-parametric AUCs were calculated via the trapezoidal rule, and 95% confidence intervals were estimated using 2000 stratified bootstrap iterations (percentile method).

### 4.8. miRNA–mRNA Analysis

For the core target genes, we performed integrated analyses using both experimental and prediction-based databases to evaluate gene–miRNA interactions. First, miRNA interaction information for each gene was collected from databases including miRTarBase, ENCORI, and TargetScan, and confidence-weighted scores were assigned based on the level of supporting evidence [75,76,77,78].

The weighting criteria were as follows. (1) In miRTarBase, +3 points were assigned if experimentally validated by any of Reporter assay, Western blot, qPCR, Microarray, NGS, pSILAC, or Other; +2 points if CLIP-Seq results were reported at least once; and +1 point if cited in two or more papers. (2) For TargetScan, +1 point was assigned when the weighted context++ score was ≤−0.20 based on the prediction value, +1 point when the probability of conservation (Pct) was ≥0.50, and +1 point when the predicted relative KD value was ≤0.50. (3) For ENCORI, +2 points were awarded if detected in CLIP-Seq experiments, +1 point if detected in degradation experiments, and +1 point if results were reported in four or more prediction algorithms (PITA, RNA22, miRmap, microT, miRanda, PicTar, TargetScan). For each miRNA–gene pair, the scores from the three databases were summed, and the confidence level was classified based on the total score. A weighted sum of 9 or higher was classified as High, 7–8 points as Moderate, and ≤6 as Low.

Subsequently, the mirBase database (https://www.mirbase.org/; accessed on 17 December 2025) was utilized to classify miRNA families and examine their associated biological processes. This analysis enabled the characterization of major miRNA groups interacting with the core target genes.

### 4.9. Molecular Docking Analysis

Structure-based blind docking simulations were performed to investigate the binding characteristics between key target genes and active compounds [34,79]. For this analysis, the top eight active compounds with high degree centrality (DC) in the PHCTP network were selected.

To prepare the target proteins, three-dimensional structure data were collected from the RCSB Protein Data Bank (PDB; https://www.rcsb.org/; accessed on 10 January 2026) using UniProt IDs. The structures were sorted by resolution, and those with the highest resolution were selected after excluding mutated structures and non-Homo sapiens entries. The selected PDB structures were then prepared by isolating the protein chains using PyMOL (ver. 3.1.6.1; Schrödinger, LLC, New York, NY, USA). In contrast, because an experimental structure was unavailable, BCL2 was modeled based on its sequence from UniProt (Accession Number P10415).

For the ligand preparation, Structure Data Files (SDF) for the selected active compounds were obtained from the PubChem database (https://pubchem.ncbi.nlm.nih.gov/; accessed on 10 January 2026) using their PubChem CIDs. These structures were preprocessed using UCSF Chimera (ver. 1.19; Resource for Biocomputing, Visualization, and Informatics, University of California, San Francisco, CA, USA) [74], which included adding hydrogen atoms via protonation at pH 7.4, assigning charges, and performing energy minimization.

Finally, blind docking simulations were executed in a 1:1 matching scheme using the CB-Dock2 server (https://cadd.labshare.cn/cb-dock2/index.php; accessed on 4 February 2026) to predict binding affinities and poses. The docking file corresponding to the binding pocket (cur pocket ID) with the lowest Vina score was selected for structural comparison with the reference complex using PyMOL (ver. 3.1.6.1) [34]. In particular, for BCL2, target docking simulations were additionally performed using the SwissDock server (http://www.swissdock.ch/; accessed on 4 February 2026) to investigate whether the active compounds bind effectively to the specific functional site identified in a previous study [80].

## 5. Conclusions

This study systematically explored the pharmacological mechanisms by which XCHT may be associated with DCI at the in silico level by integrating network pharmacology and molecular docking analysis. The analysis predicted that XCHT’s bioactive compounds may participate in the complex pathophysiology of DCI—including inflammatory responses, cell death, metabolic abnormalities, and vascular pathology—through 22 major target genes. This network ultimately converged toward key targets TLR4, BCL2, and STAT3, forming distinct axes for inflammatory immune regulation, cell survival, and signal transduction pathways. The major active compounds, stigmasterol and isorhamnetin, exhibited specific docking patterns, characterized by binding to the inactive domains of TLR4-MD-2 and STAT3, as well as the stabilizing region of BCL2. This structurally suggests the potential for XCHT to simultaneously regulate multiple targets.

In conclusion, our findings indicate that XCHT modulates inflammation, cell death, metabolism, and neuroprotection pathways related to the pathophysiology of DCI at multiple levels, theoretically supporting the network-based therapeutic potential of Korean herbal formulas. However, given the inherent limitations of in silico-based predictive analysis, future experimental validation using in vitro and in vivo DCI models is warranted to establish the biological relevance and functional significance of these findings.

## Figures and Tables

**Figure 1:**
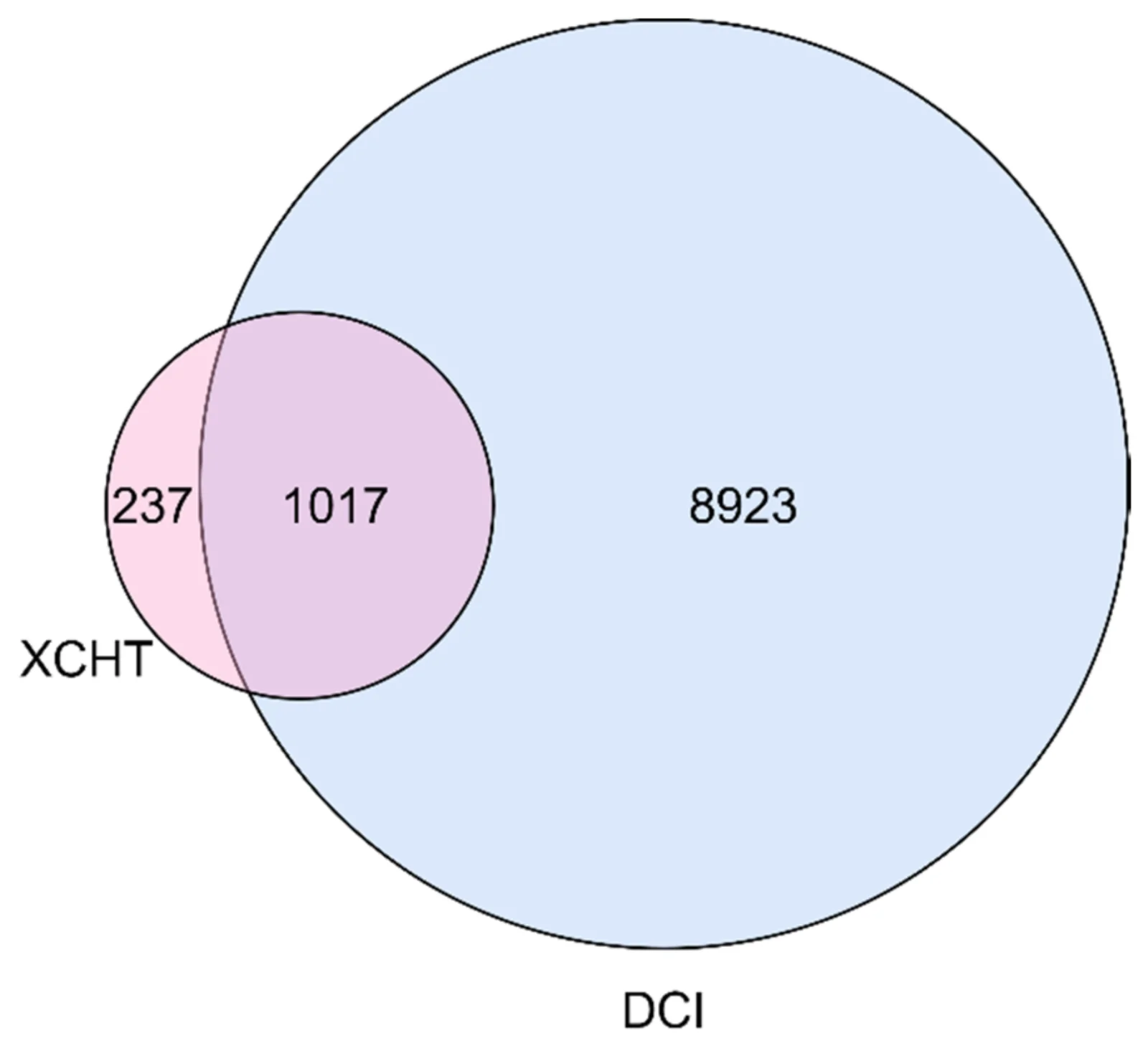
Venn diagram showing the intersecting target genes of XCHT and DCI.

**Figure 2:**
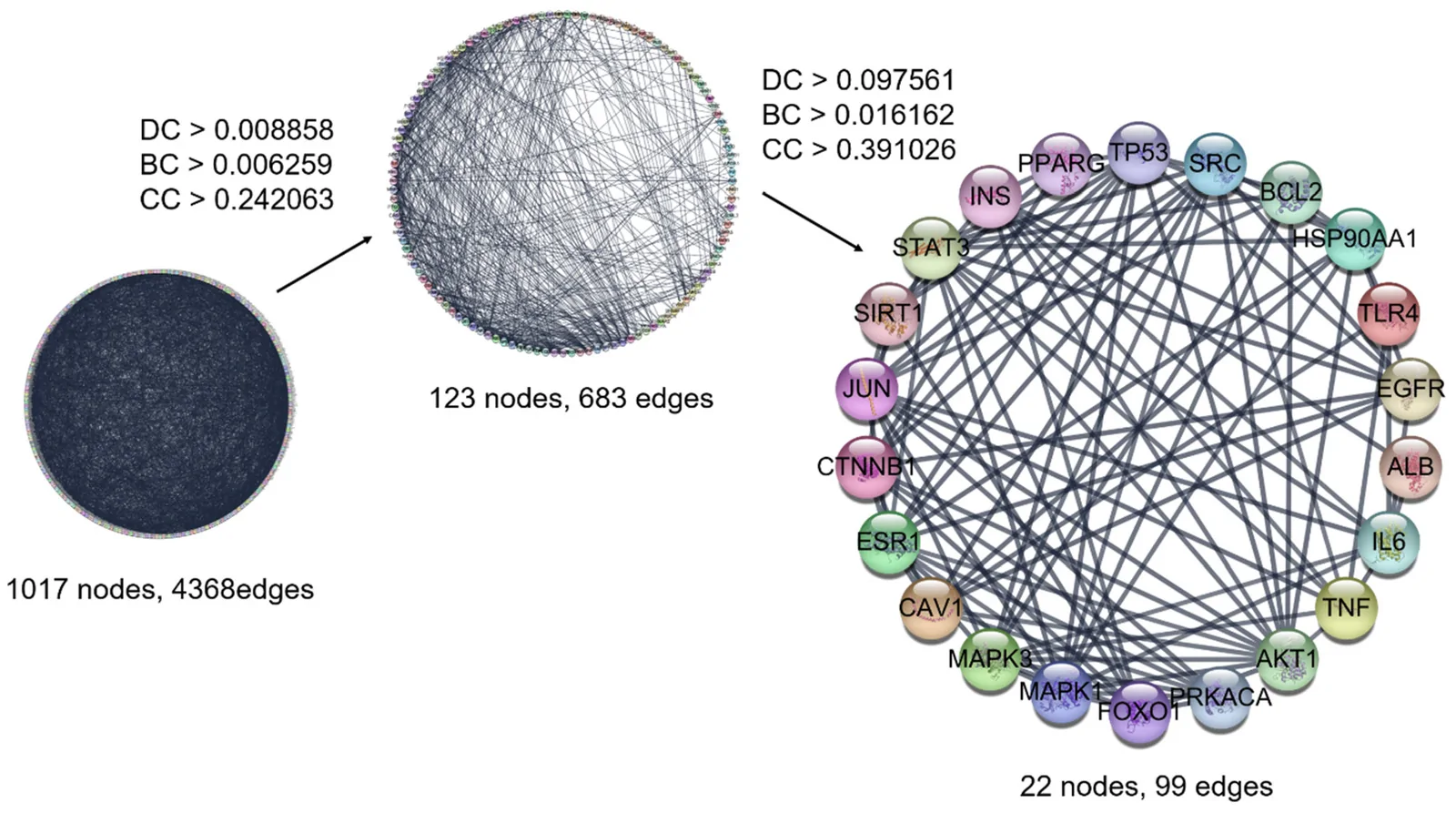
Protein–Protein Interaction (PPI) Analysis and Network Construction of Common Target Genes.

**Figure 3:**
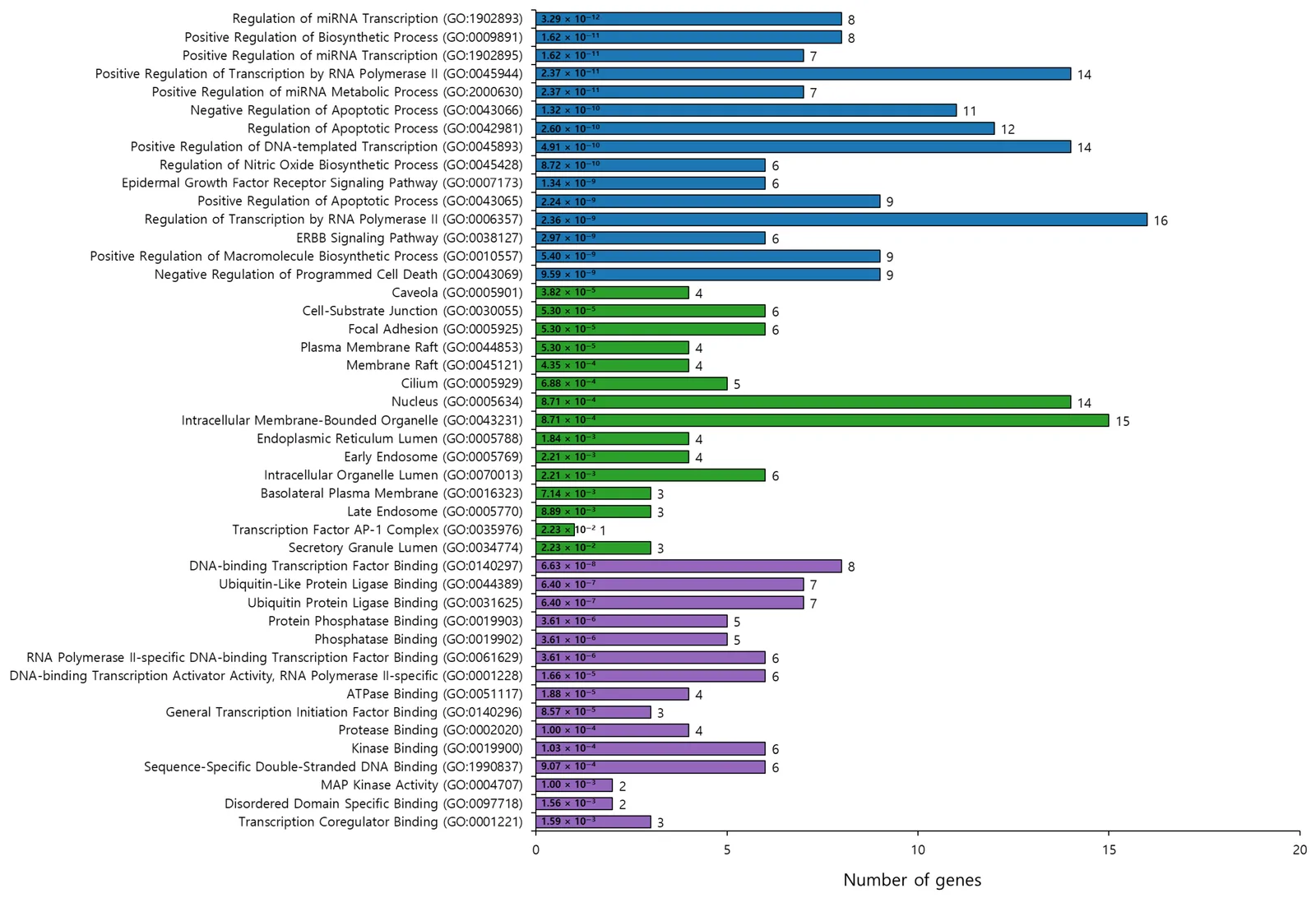
GO analysis of major target genes. Top 15 enriched GO terms for Biological Process (BP-Blue), Cellular Component (CC-Green), and Molecular Function (MF-Purple) categories, respectively, ranked by adjusted *p*-value. The bar length represents the number of genes associated with each GO term.

**Figure 4:**
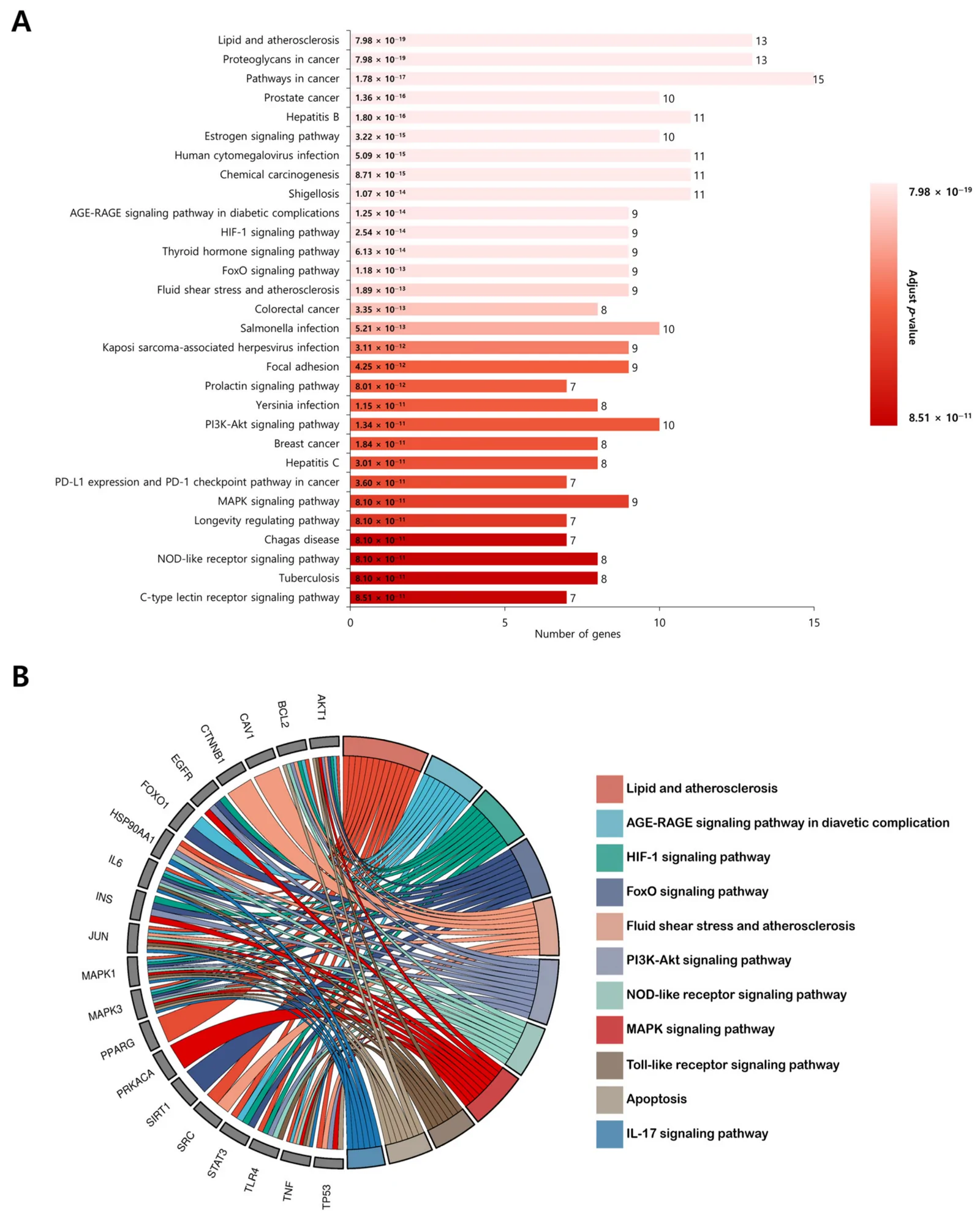
KEGG analysis of major target genes. (**A**) Top 30 KEGG pathways enriched by the 22 major target genes, ranked by adjusted *p*-value. (**B**) Network illustrating the associations between major target genes and the nine DCI-related pathways identified from enrichment analysis.

**Figure 5:**
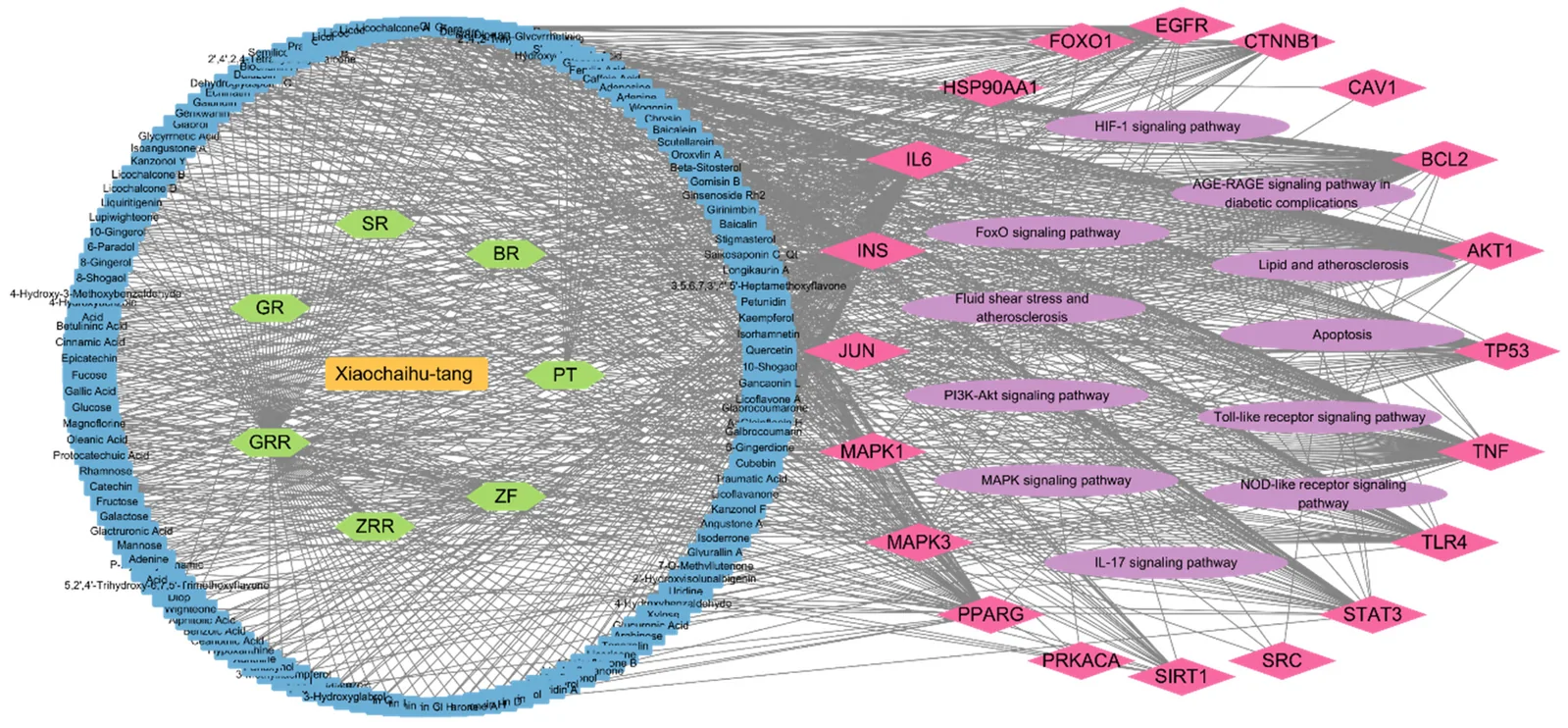
PHCTP network of XCHT-DCI.

**Figure 6:**
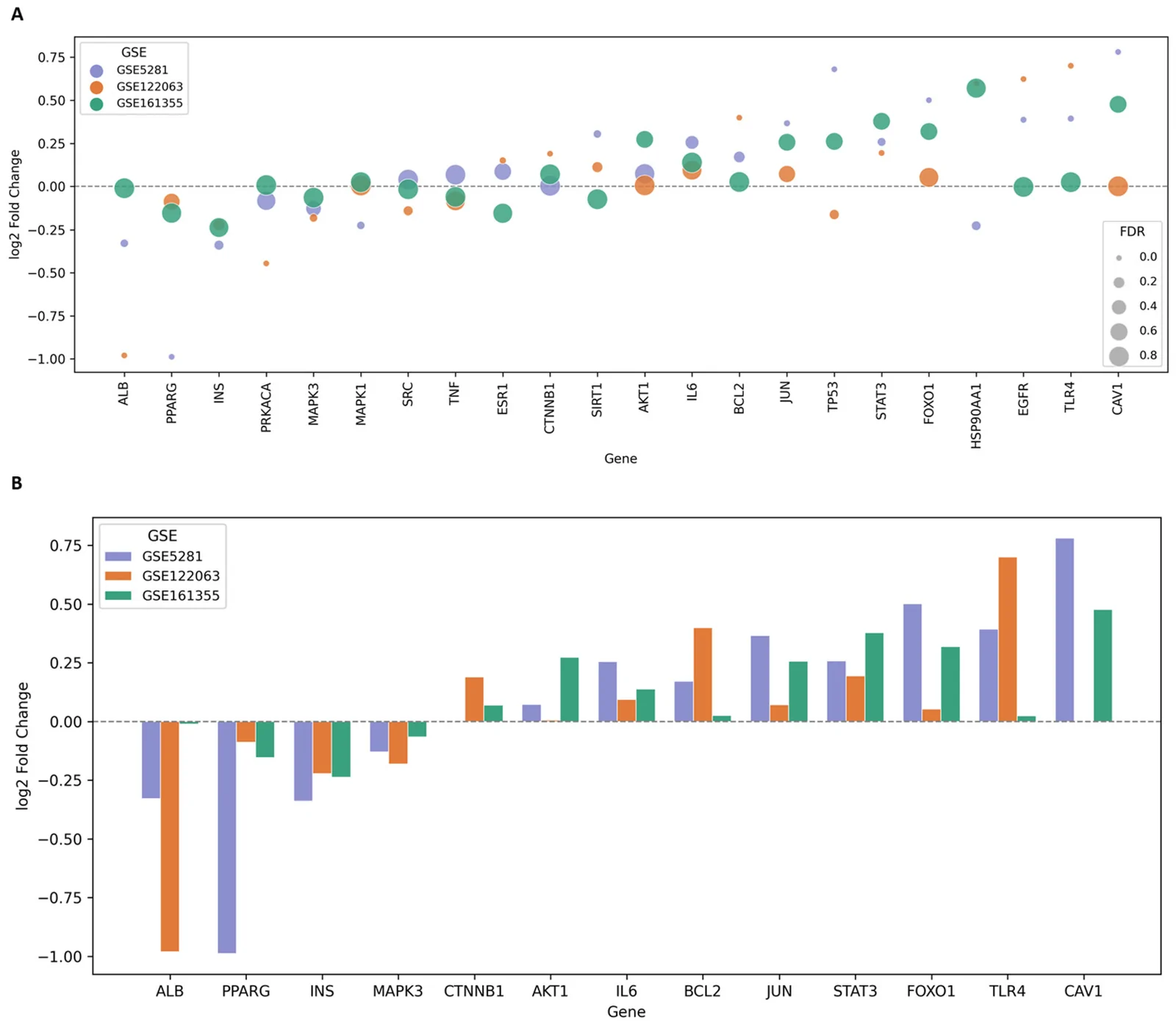
Express patterns of core target genes in GEO datasets. (**A**) Comparative dot plot of log_2_ fold change (log_2_FC) for major target genes in three independent GEO datasets. (**B**) Cross-dataset comparison of core target genes.

**Figure 7:**
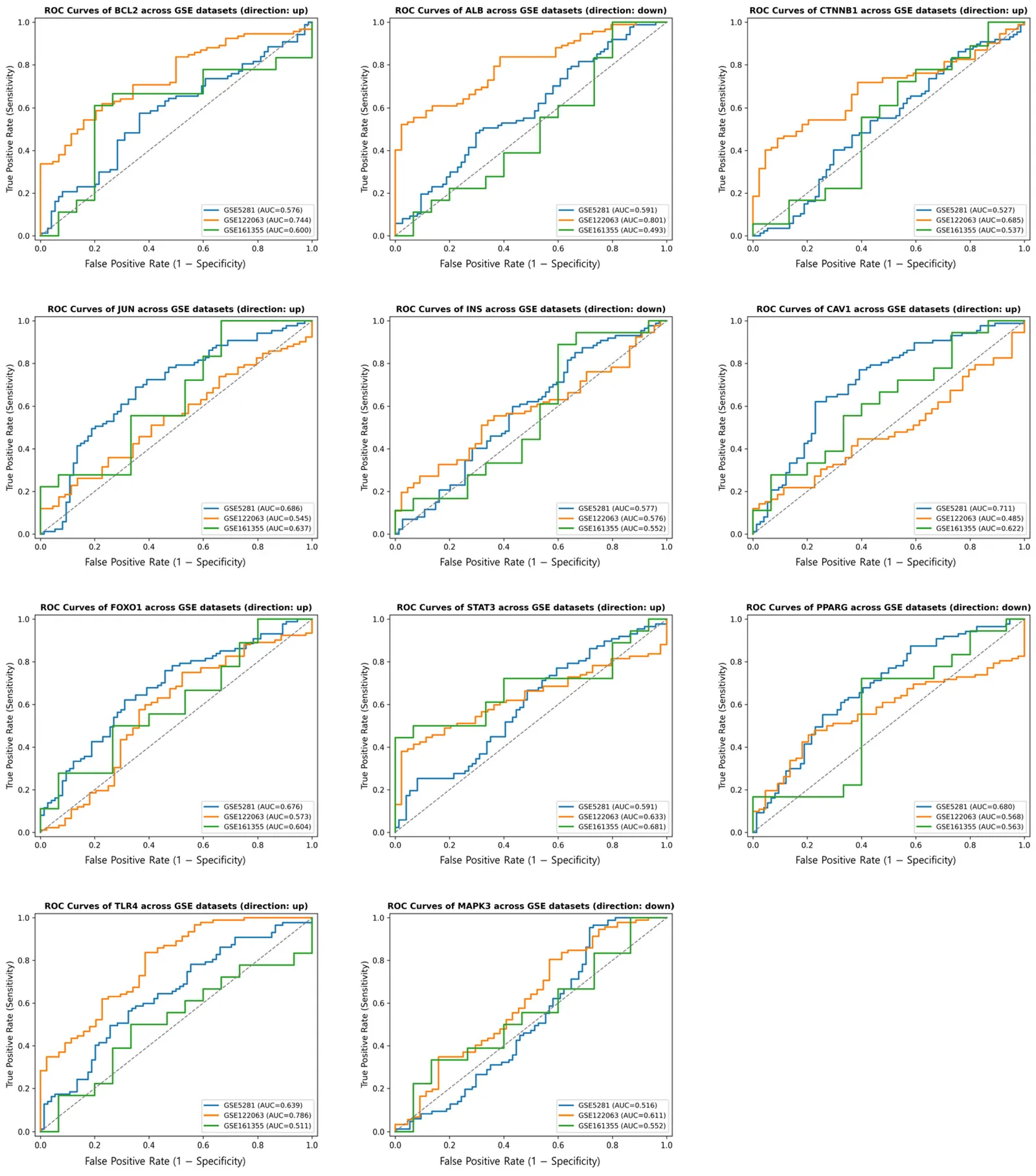
ROC curve of core target genes across GEO datasets (GSE161355-Green, GSE122063-Orange, GSE5281-Blue). The gray dashed diagonal line represents the reference line for random classification (AUC = 0.5).

**Figure 8:**
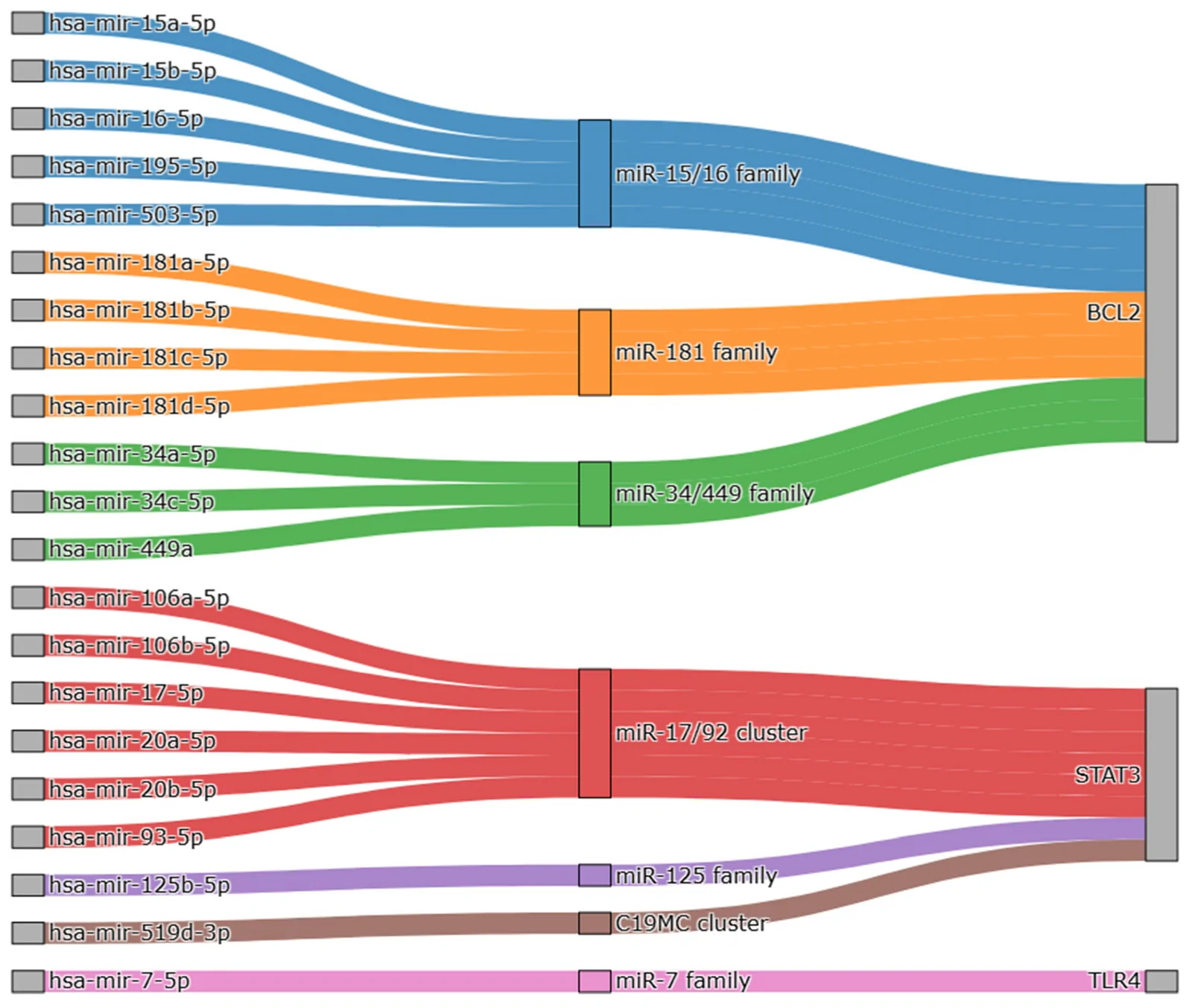
miRNA family of primary core target genes. The Sankey diagram illustrates the interactions between the primary core target genes (BCL2, STAT3, and TLR4) and their corresponding miRNA families.

**Figure 9:**
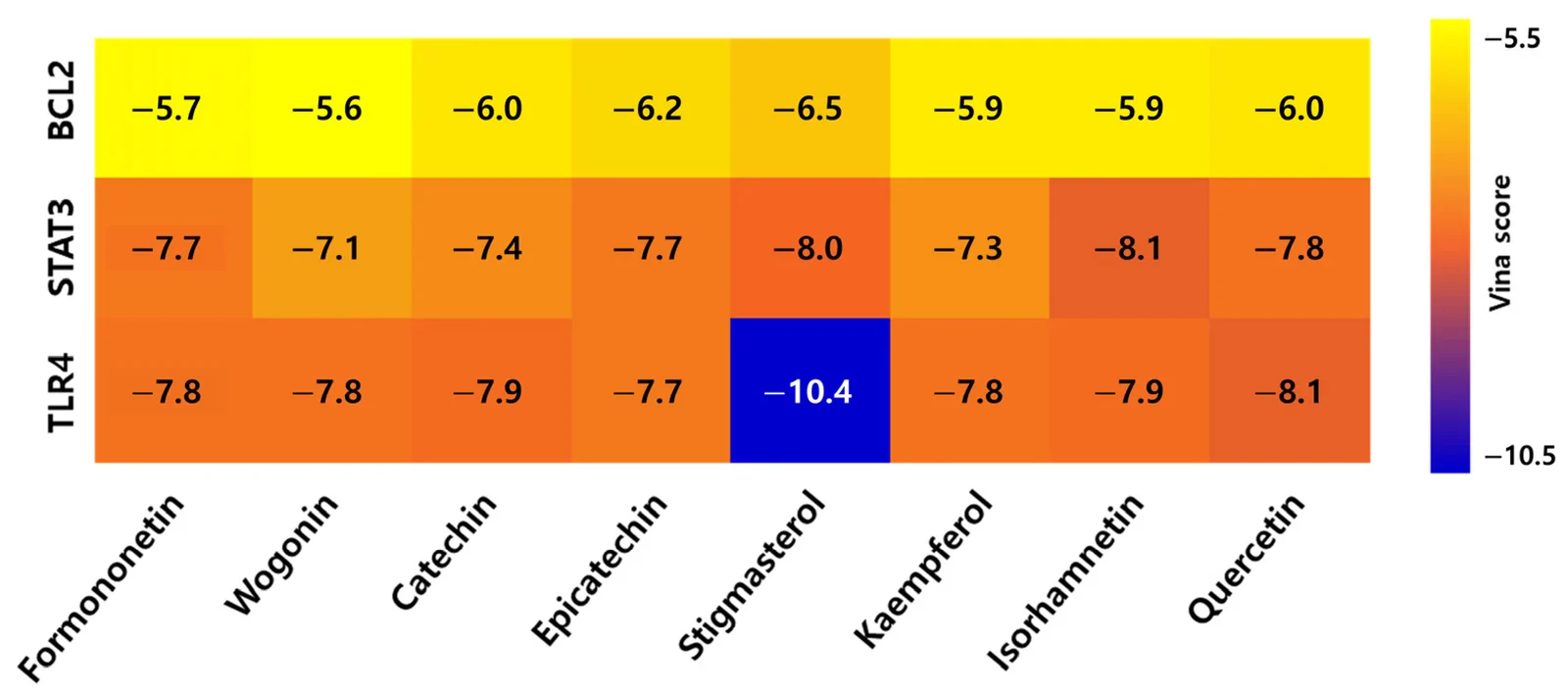
Binding affinity of eight major active compounds of XCHT with primary core target genes.

**Figure 10:**
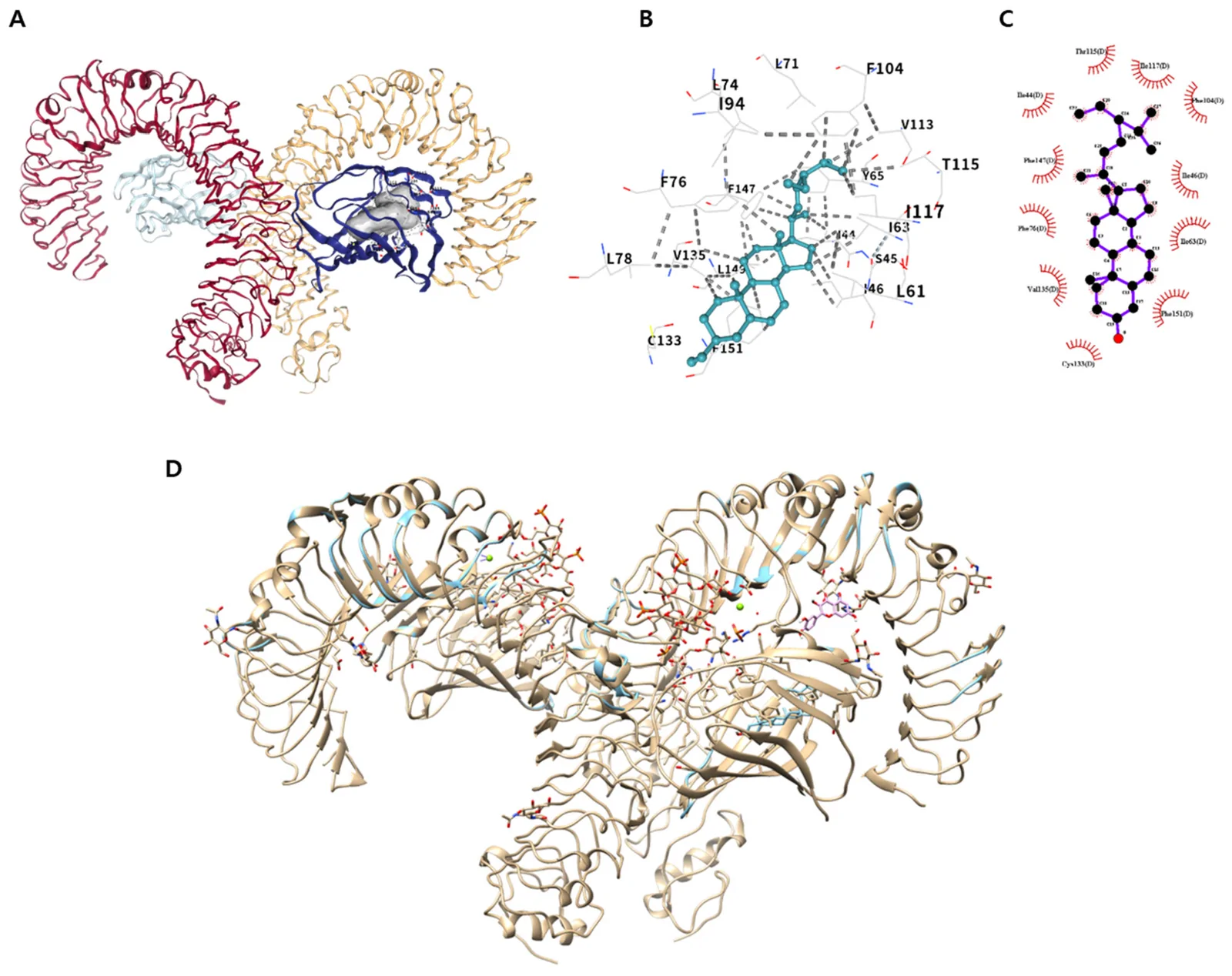
Molecular docking analysis of the TLR4 protein. (**A**) Binding pose of Stigmasterol in the TLR4 protein. (**B**) Detailed 3D view of the binding interactions. (**C**) Two-dimensional intermolecular interaction map. (**D**) Superimposed view of the docking pose with the reference complex (wheat: reference, mint: Stigmasterol).

**Table 1:** Major target genes and their degree, betweenness, and closeness centralities.

Gene Name	Degree Centrality	Betweenness Centrality	Closeness Centrality
TP53	0.319672	0.112857	0.519149
AKT1	0.295082	0.06504	0.519149
ESR1	0.270492	0.053287	0.493927
SRC	0.254098	0.049279	0.482213
STAT3	0.245902	0.032863	0.48996
CTNNB1	0.221311	0.027217	0.474708
HSP90AA1	0.213115	0.045988	0.486056
JUN	0.213115	0.01819	0.471042
MAPK1	0.204918	0.022434	0.478431
EGFR	0.188525	0.03125	0.491935
MAPK3	0.188525	0.020116	0.469231
IL6	0.180328	0.040013	0.456929
TNF	0.172131	0.030765	0.462121
PRKACA	0.155738	0.021802	0.438849
TLR4	0.155738	0.022021	0.435714
FOXO1	0.147541	0.016162	0.443636
INS	0.147541	0.056051	0.458647
BCL2	0.139344	0.020849	0.442029
PPARG	0.131148	0.040033	0.446886
SIRT1	0.131148	0.037511	0.445255
CAV1	0.122951	0.020188	0.442029
ALB	0.098361	0.033911	0.391026

**Table 2:** Selection of core target genes based on log_2_FC, FDR, and Direction.

Gene Name	GEO Series	log_2_FC	*p*-Value	FDR	Direction
AKT1	GSE161355	0.2727	0.145227	0.561718	up
	GSE122063	0.0057	0.871387	0.958525	up
	GSE5281	0.073	0.655579	0.759092	up
ALB	GSE161355	−0.011	0.948311	0.973548	down
	GSE122063	−0.9801	4.40 × 10^−12^	4.85 × 10^−11^	down
	GSE5281	−0.3284	0.023133	0.055906	down
BCL2	GSE161355	0.0254	0.844143	0.973548	up
	GSE122063	0.3988	1.88 × 10^−7^	0.000001	up
	GSE5281	0.1707	0.121234	0.190511	up
CAV1	GSE161355	0.4765	0.153196	0.561718	up
	GSE122063	0.0003	0.997644	0.997644	up
	GSE5281	0.7806	0.000017	0.000279	up
CTNNB1	GSE161355	0.0698	0.773167	0.973548	up
	GSE122063	0.1896	0.000109	0.000343	up
	GSE5281	0.0034	0.973280	0.973280	up
FOXO1	GSE161355	0.3186	0.147327	0.561718	up
	GSE122063	0.0523	0.622689	0.721008	up
	GSE5281	0.5007	0.000074	0.000409	up
IL6	GSE161355	0.1384	0.428512	0.857023	up
	GSE122063	0.0938	0.594437	0.721008	up
	GSE5281	0.2553	0.209982	0.307973	up
INS	GSE161355	−0.237	0.262724	0.722492	down
	GSE122063	−0.2215	0.134460	0.211294	down
	GSE5281	−0.3395	0.068535	0.115982	down
JUN	GSE161355	0.2561	0.148094	0.561718	up
	GSE122063	0.0717	0.378173	0.519988	up
	GSE5281	0.3664	0.002710	0.009936	up
MAPK3	GSE161355	−0.0657	0.732613	0.973548	down
	GSE122063	−0.1818	0.024729	0.054403	down
	GSE5281	−0.129	0.302048	0.415316	down
PPARG	GSE161355	−0.154	0.335855	0.738880	down
	GSE122063	−0.089	0.339836	0.498426	down
	GSE5281	−0.9882	0.000046	0.000338	down
STAT3	GSE161355	0.3779	0.027361	0.561718	up
	GSE122063	0.1938	0.001246	0.003425	up
	GSE5281	0.258	0.035344	0.070689	up
TLR4	GSE161355	0.0242	0.870266	0.973548	up
	GSE122063	0.7004	5.61 × 10^−9^	4.12 × 10^−8^	up
	GSE5281	0.3937	0.003330	0.010465	up

**Table 3:** Meta-analysis of candidate gene significance across three GEO datasets.

Gene Name	Fisher’s χ^2^	Combined *p*-Value	Benjamini–Hochberg FDR
ALB	64.09	6.60 × 10^−12^	8.58 × 10^−11^
TLR4	53.84	7.93 × 10^−10^	5.16 × 10^−9^
BCL2	39.70	5.23 × 10^−7^	2.27 × 10^−6^
STAT3	31.42	2.11 × 10^−5^	6.86 × 10^−5^
CAV1	29.91	4.10 × 10^−5^	1.07 × 10^−4^
PPARG	28.47	7.66 × 10^−5^	1.66 × 10^−4^
FOXO1	27.95	9.60 × 10^−5^	1.78 × 10^−4^
CTNNB1	22.97	8.06 × 10^−4^	1.31 × 10^−3^
JUN	21.75	1.35 × 10^−3^	1.95 × 10^−3^
INS	16.21	1.27 × 10^−2^	1.65 × 10^−2^
MAPK3	14.58	2.38 × 10^−2^	2.82 × 10^−2^
IL6	10.02	1.24 × 10^−1^	1.34 × 10^−1^
AKT1	9.14	1.66 × 10^−1^	1.66 × 10^−1^

**Table 4:** ROC–AUC analysis of core target genes across the three GEO series datasets.

Gene Name	AUC Value			
	GSE161355	GSE122063	GSE5281	Mean
AKT1	0.659	0.519	0.533	0.57
ALB	0.493	0.801	0.591	0.628
BCL2	0.6	0.744	0.576	0.64
CAV1	0.622	0.485	0.711	0.606
CTNNB1	0.537	0.685	0.527	0.583
FOXO1	0.604	0.573	0.676	0.618
IL6	0.622	0.504	0.569	0.565
INS	0.552	0.576	0.577	0.568
JUN	0.637	0.545	0.686	0.623
MAPK3	0.552	0.611	0.516	0.56
PPARG	0.563	0.568	0.68	0.604
STAT3	0.681	0.633	0.591	0.635
TLR4	0.511	0.786	0.639	0.645

**Table 5:** Predicted miRNA–mRNA interactions for primary core target genes and their database-derived weight scores.

Gene	miRNA	miRNA Family	Weight Scores			
			Total	miRTarBase	TargetScan	ENCORI
BCL2	hsa-mir-15a-5p	miR-15/16	9	4	2	3
BCL2	hsa-mir-15b-5p	miR-15/16	9	4	2	3
BCL2	hsa-mir-16-5p	miR-15/16	9	4	2	3
BCL2	hsa-mir-181a-5p	miR-181	9	4	2	3
BCL2	hsa-mir-181b-5p	miR-181	9	4	2	3
BCL2	hsa-mir-181c-5p	miR-181	9	4	2	3
BCL2	hsa-mir-181d-5p	miR-181	9	4	2	3
BCL2	hsa-mir-195-5p	miR-15/16	9	4	2	3
BCL2	hsa-mir-34a-5p	miR-34/449	9	4	2	3
BCL2	hsa-mir-34c-5p	miR-34/449	9	4	2	3
BCL2	hsa-mir-449a	miR-34/449	9	4	2	3
BCL2	hsa-mir-503-5p	miR-15/16	9	4	2	3
STAT3	hsa-mir-106a-5p	miR-17/92	9	4	2	3
STAT3	hsa-mir-106b-5p	miR-17/92	9	4	2	3
STAT3	hsa-mir-125b-5p	miR-125	9	4	2	3
STAT3	hsa-mir-17-5p	miR-17/92	9	4	2	3
STAT3	hsa-mir-20a-5p	miR-17/92	9	4	2	3
STAT3	hsa-mir-20b-5p	miR-17/92	9	4	2	3
STAT3	hsa-mir-519d-3p	C19MC	9	4	2	3
STAT3	hsa-mir-93-5p	miR-17/92	9	4	2	3
TLR4	hsa-mir-7-5p	miR-7	9	6	0	3

**Table 6:** Docking results of primary core target gene and compound complex.

Primary Core Target Gene and Compound Complex	Vina Score	Center (x, y, z)	Docking Size (x, y, z)
TLR4 + Stigmasterol	−10.4	−7, −8, −26	25, 25, 32

**Table 7:** Primary core target gene–compound complexes and corresponding reference complexes.

Primary Core Target Gene and Compound Complex	Reference
TLR4 + Stigmasterol	TLR4-3FXI + MD-2

**Table 8:** Summary of the GEO series datasets used for analysis.

GSE161355	
Title	Type 2 Diabetes Mellitus is Associated with Transcriptome Alterations in Cortical Neurons and Associated Neurovascular Unit Cells in the Ageing Brain
Platform	GPL570 ([HG-U133_Plus_2] Affymetrix Human Genome U133 Plus 2.0 Array)
Organism	*Homo sapiens*
Samples	case-18/control-15 (ratio-1.2:1)
Source	Temporal lobe tissue
Study description	Gene expression profiling of temporal lobe samples from patients with type 2 diabetes mellitus combined with cognitive impairment and aging, compared to normal controls.
GSE122063	
Title	Dementia Comparison: VaD vs. AD vs. Controls
Platform	GPL16699 (Agilent-039494 SurePrint G3 Human GE v2 8x60K Microarray)
Organism	*Homo sapiens*
Samples	case-92/control-44 (ratio-2.09:1)
Source	Temporal lobe autopsy tissue
Study description	Microarray analysis of temporal lobe samples from Alzheimer’s disease patients and non-demented controls to identify differentially expressed genes related to cognitive decline and neurodegeneration.
GSE5281	
Title	Alzheimer’s disease and the normal aged brain (steph-affy-human-433773)
Platform	GPL570 ([HG-U133_Plus_2] Affymetrix Human Genome U133 Plus 2.0 Array)
Organism	*Homo sapiens*
Samples	case-87/control-74 (ratio-1.18:1)
Source	Hippocampus, entorhinal cortex, prefrontal cortex, and temporal cortex
Study description	Gene expression profiling across multiple brain regions from Alzheimer’s disease patients and matched non-AD controls, designed to explore regional variation in neurodegenerative gene signatures.

## Data Availability

The raw data supporting the conclusions of this article will be made available by the authors on request.

## Supplementary Materials

The following supporting information can be downloaded at https://www.mdpi.com/article/10.3390/brainsci16101005/s1. Table S1: Physicochemical and Pharmacokinetic Properties of Active Compounds (OASIS); Table S2: Physicochemical and Pharmacokinetic Properties of Active Compounds (TCMSP); Table S3: List of the common genes of XCHT and DCI; Table S4: Top15 GO Biological Process Enrichment of Major Target Genes; Table S5: Top15 GO Cellular Component of Major Target Genes; Table S6: Top15 GO Molecular function Enrichment of Major Target Genes; Table S7: Top 30 KEGG pathway Enrichment of Major Target Genes.
