# Supplementary material for: Investigating the Pharmacological Basis of Xiaochaihu-Tang in Diabetic Cognitive Impairment: A Network Pharmacology Approach
Source: Brain Sci. 2026 Sep 23;16(10):1005. doi: 10.3390/brainsci16101005 (PMC13702502; doi:10.3390/brainsci16101005)
Supplement: Supplementary file 1 [file brainsci-16-01005-s001.zip › brainsci-4528144-supplementary.pdf]

Supplemental Table S1. Physicochemical and Pharmacokinetic Properties of Active Compounds (OASIS).

Compound	CID	Molecular Formula	Canonical SMILES	Molecular weight	H-bond acceptors	H-bond donors	MlogP	Lipinski violations	TPSA	Bioavailability score	Herb
β-Ecdysone	5459840	C27H44O7	CC12CCC3C(=CC(=O)C4C3(CC(C(C4)O)O)C)C1(CCC2C(C)(C(CCC(C)(C)O)O)O)O	480.63	7	6	1.13	1	138.45	0.55	Ginseng Radix
Panaxynol	5469789	C17H24O	CCCCCCCC=CCC#CC#CC(C=C)O	244.37	1	1	4.26	1	20.23	0.55	Ginseng Radix
Kanzonol Y	10001604	C25H30O5	CC(=CCC1=CC(=C(C=C1O)O)C(=O)C(CC2=CC(=C(C=C2)O)CC=C(C)C)O)C	410.5	5	4	2.95	0	97.99	0.55	Glycyrrhizae Radix et Rhizoma
Glicophenone	10021298	C20H22O6	CC(=CCC1=C(C(=C(C=C1O)O)CC(=O)C2=C(C=C(C=C2)O)O)OC)C	358.39	6	4	1.67	0	107.22	0.55	Glycyrrhizae Radix et Rhizoma
Licoarylcoumarin	10090416	C21H20O6	CC(C)(C=C)C1=C2C(=C(C=C1O)OC)C=C(C(=O)O2)C3=C(C=C(C=C3)O)O	368.38	6	3	2.13	0	100.13	0.55	Glycyrrhizae Radix et Rhizoma
2',4',2,4-Tetrahydroxychalcone	10107266	C15H12O5	C1=CC(=C(C=C1O)O)C=CC(=O)C2=C(C=C(C=C2)O)O	272.25	5	4	1.02	0	97.99	0.55	Glycyrrhizae Radix et Rhizoma
Dehydroglyasperi	10109594	C22H24O	CC(=CCC1=C(C=C2C(=C1OC)C=C	368.42	5	2	2.53	0	68.15	0.55	Glycyrrhizae

n D		5	(CO2)C3=C(C=C(C=C3)O)O)OC)C								Radix et Rhizoma
18β-Glycyrrhetinic acid	10114	C30H46O4	CC1(C2CCC3(C(C2(CCC1O)C)C(=O)C=C4C3(CCC5(C4CC(CC5)(C)C(=O)O)C)C)C)C	470.68	4	2	4.87	1	74.6	0.85	Glycyrrhizae Radix et Rhizoma
Kanzonol F	101666840	C26H28O5	CC(=CCC1=C(C2=C(C=C1O)OCC3C2OC4=C3C=C5C=CC(OC5=C4)(C)C)OC)C	420.5	5	1	3.37	0	57.15	0.55	Glycyrrhizae Radix et Rhizoma
Kanzonol V	102444980	C24H24O4	CC(=CCC1=C(C=C2C(=C1)C=C(O2)C3=C4C(=C(C=C3)O)C=CC(O4)(C)C)O)C	376.44	4	2	3.37	0	62.83	0.55	Glycyrrhizae Radix et Rhizoma
Licoflavone C	10246505	C20H18O5	CC(=CCC1=C2C(=C(C=C1O)O)C(=O)C=C(O2)C3=CC=C(C=C3)O)C	338.35	5	3	1.64	0	90.9	0.55	Glycyrrhizae Radix et Rhizoma
1-Methoxyphaseollin	10247433	C21H20O5	CC1(C=CC2=C(O1)C=CC3=C2OC4C3COC5=C4C(=CC(=C5)O)OC)C	352.38	5	1	2.39	0	57.15	0.55	Glycyrrhizae Radix et Rhizoma
Isoglabrone	102597283	C20H16O5	CC1(C=CC2=C(O1)C=CC3=C2OC=C(C3=O)C4=C(C=C(C=C4)O)O)C	336.34	5	2	1.64	0	79.9	0.55	Glycyrrhizae Radix et Rhizoma
Glicoricone	10361658	C21H20O	CC(=CCC1=C(C(=C(C=C1O)O)C2=	368.38	6	3	1.31	0	100.13	0.55	Glycyrrhizae

		6	COC3=C(C2=O)C=CC(=C3)O)OC)C								Radix et Rhizoma
Allolicoisoflavone B	10383349	C20H16O6	CC1(C=CC2=C(C=CC(=C2O1)C3=COC4=CC(=CC(=C4C3=O)O)O)O)O)C	352.34	6	3	1.09	0	100.13	0.55	Glycyrrhizae Radix et Rhizoma
Licochalcone D	10473311	C21H22O5	CC(=CCC1=C(C=CC(=C1)C(=O)C=CC2=C(C(=C(C=C2)O)O)OC)O)C	354.4	5	3	2.36	0	86.99	0.55	Glycyrrhizae Radix et Rhizoma
Glabrocoumarone A=glainflanin H	10542808	C19H16O4	CC1(C=CC2=C(C=CC(=C2O1)C3=CC4=C(O3)C=C(C=C4)O)O)C	308.33	4	2	2.35	0	62.83	0.55	Glycyrrhizae Radix et Rhizoma
Glycyrrhiza-isoflavone C	10546844	C21H18O6	CC1(C=CC2=C(O1)C(=CC(=C2)C3=COC4=C(C3=O)C(=CC(=C4)O)OC)O)C	366.36	6	2	1.31	0	89.13	0.55	Glycyrrhizae Radix et Rhizoma
Licoleafol	11111496	C20H20O7	CC(=CCC1=C2C(=C(C=C1O)O)C(=O)CC(O2)C3=CC(=C(C=C3)O)O)CO	372.37	7	5	0.48	0	127.45	0.55	Glycyrrhizae Radix et Rhizoma
Licoflavone B	11349817	C25H26O4	CC(=CCC1=CC2=C(C=C1O)OC(=CC2=O)C3=CC(=C(C=C3)O)CC=C(C)C)C	390.47	4	2	3.2	0	70.67	0.55	Glycyrrhizae Radix et Rhizoma
Glabroisoflavanon	11405466	C21H20O	CC1(C=CC2=C(O1)C=CC3=C2OC	352.38	5	1	2.04	0	64.99	0.55	Glycyrrhizae

e		5	C(C3=O)C4=C(C=C(C=C4)OC)O)C								Radix et Rhizoma
Galbrocoumarin	11427657	C20H16O5	CC1(C=CC2=C(C=CC(=C2O1)C3=CC4=C(C=C(C=C4)O)OC3=O)O)C	336.34	5	2	2.45	0	79.9	0.55	Glycyrrhizae Radix et Rhizoma
Liquiritigenin	114829	C15H12O4	C1C(OC2=C(C1=O)C=CC(=C2)O)C3=CC=C(C=C3)O	256.25	4	2	1.27	0	66.76	0.55	Glycyrrhizae Radix et Rhizoma
Glabrol	11596309	C25H28O4	CC(=CCC1=C(C=CC(=C1)C2CC(=O)C3=C(O2)C(=C(C=C3)O)CC=C(C)C)O)C	392.49	4	2	3.38	0	66.76	0.55	Glycyrrhizae Radix et Rhizoma
Licopyranocoumarin	122851	C21H20O7	CC1(CCC2=C(O1)C=C3C(=C2OC)C=C(C(=O)O3)C4=C(C=C(C=C4)O)O)CO	384.38	7	3	1.4	0	109.36	0.55	Glycyrrhizae Radix et Rhizoma
11-Deoxoglycyrrhetinic acid	12305517	C30H48O3	CC1(C2CCC3(C(C2(CCC1O)C)CC=C4C3(CCC5(C4CC(CC5)(C)C(=O)O)C)C)C)C	456.7	3	2	5.82	1	57.53	0.85	Glycyrrhizae Radix et Rhizoma
Glabranin	124049	C20H20O4	CC(=CCC1=C2C(=C(C=C1O)O)C(=O)CC(O2)C3=CC=CC=C3)C	324.37	4	2	2.38	0	66.76	0.55	Glycyrrhizae Radix et Rhizoma
Isoglycyrol	124050	C21H18O	CC1(CCC2=C(O1)C=C3C(=C2OC)	366.36	6	1	2.36	0	82.04	0.55	Glycyrrhizae

		6	C4=C(C5=C(O4)C=C(C=C5)O)C(=O)O3)C								Radix et Rhizoma
Galbridin	124052	C20H20O4	CC1(C=CC2=C(O1)C=CC3=C2OCC(C3)C4=C(C=C(C=C4)O)O)C	324.37	4	2	2.73	0	58.92	0.55	Glycyrrhizae Radix et Rhizoma
Licobenzofuran	133867	C21H22O5	CC(=CCC1=C(C=CC(=C1)C2=C(C3=CC(=C(C=C3O2)OC)OC)O)O)C	354.4	5	2	2.24	0	72.06	0.55	Glycyrrhizae Radix et Rhizoma
Glycyfuranocoumarin A	139200316	C21H20O6	CC1C(C2=C3C(=C(C=C2O1)OC)C=C(C(=O)O3)C4=C(C=C(C=C4)O)O)(C)C	368.38	6	2	2.2	0	89.13	0.55	Glycyrrhizae Radix et Rhizoma
Isoglycycoumarin	14187587	C21H20O6	CC1(CCC2=C(O1)C=C3C(=C2OC)C=C(C(=O)O3)C4=C(C=C(C=C4)O)O)C	368.38	6	2	2.2	0	89.13	0.55	Glycyrrhizae Radix et Rhizoma
Licoflavanone	14218028	C20H20O5	CC(=CCC1=C(C=CC(=C1)C2CC(=O)C3=C(C=C(C=C3O2)O)O)O)C	340.37	5	3	1.82	0	86.99	0.55	Glycyrrhizae Radix et Rhizoma
2'-Hydroxyisolupalbigenin	14237659	C25H26O6	CC(=CCC1=C(C=CC(=C1O)C2=COC3=C(C(=CC(=C3C2=O)O)O)CC=C(C)C)O)C	422.47	6	4	2.09	0	111.13	0.55	Glycyrrhizae Radix et Rhizoma
Isoderrone	14237660	C20H16O	CC1(C=CC2=C(O1)C=CC(=C2)C3=	336.34	5	2	1.64	0	79.9	0.55	Glycyrrhizae

		5	COC4=CC(=CC(=C4C3=O)O)O)C								Radix et Rhizoma
Gancaonin L	14604077	C20H18O6	CC(=CCC1=C2C(=C(C=C1O)O)C(=O)C(=CO2)C3=CC(=C(C=C3)O)O)C	354.35	6	4	1.09	0	111.13	0.55	Glycyrrhizae Radix et Rhizoma
Hispaglabridin B	15228661	C25H26O4	CC1(C=CC2=C(O1)C=CC(=C2O)C3CC4=C(C5=C(C=C4)OC(C=C5)(C)C)OC3)C	390.47	4	1	3.73	0	47.92	0.55	Glycyrrhizae Radix et Rhizoma
Glabrocoumarone B	15233562	C19H16O4	CC1(C=CC2=C(O1)C=CC(=C2O)C3=CC4=C(O3)C=C(C=C4)O)C	308.33	4	2	2.35	0	62.83	0.55	Glycyrrhizae Radix et Rhizoma
Angustone A	15664151	C25H26O6	CC(=CCC1=C(C=CC(=C1O)C2=COC3=C(C2=O)C(=C(C(=C3)O)CC=C(C)C)O)O)C	422.47	6	4	2.09	0	111.13	0.55	Glycyrrhizae Radix et Rhizoma
Glyurallin A	15818598	C21H20O5	CC(=CCC1=C(C2=C(C=C1O)OCC3=C2OC4=C3C=CC(=C4)O)OC)C	352.38	5	2	2.24	0	72.06	0.55	Glycyrrhizae Radix et Rhizoma
Glycyuralin E	163184220	C21H22O6	CC1(CCC2=C(O1)C=C(C(=C2OC)C3=C(C4=C(O3)C=C(C=C4)O)CO)O)C	370.4	6	3	1.5	0	92.29	0.55	Glycyrrhizae Radix et Rhizoma
Isoangustone A	21591148	C25H26O	CC(=CCC1=C(C(=CC(=C1)C2=CO	422.47	6	4	2.09	0	111.13	0.55	Glycyrrhizae

		6	C3=C(C2=O)C(=C(C(=C3)O)CC=C(C)C)O)O)O)C								Radix et Rhizoma
Isolupalbigenin	26238934	C25H26O5	CC(=CCC1=C(C=CC(=C1)C2=COC3=C(C(=CC(=C3C2=O)O)O)CC=C(C)C)O)C	406.47	5	3	2.64	0	90.9	0.55	Glycyrrhizae Radix et Rhizoma
Glycyrrhetic acid	3230	C30H46O4	CC1(C2CCC3(C(C2(CCC1O)C)C(=O)C=C4C3(CCC5(C4CC(CC5)(C)C(=O)O)C)C)C)C	470.68	4	2	4.87	1	74.6	0.85	Glycyrrhizae Radix et Rhizoma
Licoisoflavanone	392443	C20H18O6	CC1(C=CC2=C(O1)C=CC(=C2O)C3COC4=CC(=CC(=C4C3=O)O)O)C	354.35	6	3	1.28	0	96.22	0.55	Glycyrrhizae Radix et Rhizoma
Glyinflanin A	42607642	C25H28O5	CC(=CCC1=CC(=C(C=C1O)O)C(=O)C=C(C2=CC(=C(C=C2)O)CC=C(C)C)O)C	408.49	5	4	2.87	0	97.99	0.56	Glycyrrhizae Radix et Rhizoma
7-O-Methyllutenone	441251	C21H20O6	CC(=CCC1=C(C=C2C(=C1O)C(=O)C(=CO2)C3=C(C=C(C=C3)O)O)OC)C	368.38	6	3	1.31	0	100.13	0.55	Glycyrrhizae Radix et Rhizoma
Hispaglabridin A	442774	C25H28O4	CC(=CCC1=C(C=CC(=C1O)C2CC3=C(C4=C(C=C3)OC(C=C4)(C)C)OC2)O)C	392.49	4	2	3.73	0	58.92	0.55	Glycyrrhizae Radix et Rhizoma
Ononin	442813	C22H22O	COC1=CC=C(C=C1)C2=COC3=C(	430.4	9	4	-0.89	0	138.82	0.55	Glycyrrhizae

		9	C2=O)C=CC(=C3)OC4C(C(C(C(O4)CO)O)O)O								Radix et Rhizoma
11b-Hydroxy-11b,1-dihydromedicarpin	44437741	C16H16O5	COC1=CC2=C(C=C1)C3COC4=CC(=O)CCC4(C3O2)O	288.3	5	1	0.58	0	64.99	0.85	Glycyrrhizae Radix et Rhizoma
Licochalcone E	46209991	C21H22O4	CC(C1=C(C=C(C(=C1)C=CC(=O)C2=CC=C(C=C2)O)OC)O)C(=C)C	338.4	4	2	2.92	0	66.76	0.55	Glycyrrhizae Radix et Rhizoma
Dehydroglyasperin C	480775	C21H22O5	CC(=CCC1=C(C2=C(C=C1O)OCC(=C2)C3=C(C=C(C=C3)O)O)OC)C	354.4	5	3	2.31	0	79.15	0.55	Glycyrrhizae Radix et Rhizoma
Gancaonin I	480777	C21H22O5	CC(=CCC1=C(C=C2C(=C1OC)C=C(O2)C3=C(C=C(C=C3)O)O)OC)C	354.4	5	2	2.24	0	72.06	0.55	Glycyrrhizae Radix et Rhizoma
Gancaonin G	480780	C21H20O5	CC(=CCC1=C(C=C2C(=C1O)C(=O)C(=CO2)C3=CC=C(C=C3)O)OC)C	352.38	5	2	1.86	0	79.9	0.55	Glycyrrhizae Radix et Rhizoma
6,8-Diprenylgenistein	480783	C25H26O5	CC(=CCC1=C(C(=C2C(=C1O)C(=O)C(=CO2)C3=CC=C(C=C3)O)CC=C(C)C)O)C	406.47	5	3	2.64	0	90.9	0.55	Glycyrrhizae Radix et Rhizoma

Glycyrin	480787	C22H22O6	CC(=CCC1=C(C=C2C(=C1OC)C=C(C(=O)O2)C3=C(C=C(C=C3)O)O)OC)C	382.41	6	2	2.34	0	89.13	0.55	Glycyrrhizae Radix et Rhizoma
3-Hydroxyglabrol	480854	C25H28O5	CC(=CCC1=C(C=CC(=C1)C2C(C(=O)C3=C(O2)C(=C(C=C3)O)CC=C(C)C)O)O)C	408.49	5	3	2.55	0	86.99	0.55	Glycyrrhizae Radix et Rhizoma
Glyasperin C	480859	C21H24O5	CC(=CCC1=C(C2=C(C=C1O)OCC(C2)C3=C(C=C(C=C3)O)O)OC)C	356.41	5	3	2.39	0	79.15	0.55	Glycyrrhizae Radix et Rhizoma
Glyasperin D	480860	C22H26O5	CC(=CCC1=C(C=C2C(=C1OC)CC(CO2)C3=C(C=C(C=C3)O)O)OC)C	370.44	5	2	2.61	0	68.15	0.55	Glycyrrhizae Radix et Rhizoma
Kanzonol H	480863	C26H32O5	CC(=CCC1=C(C=CC(=C1O)C2CC3=C(C=C4C(=C3OC)CCC(O4)(C)C)OC2)O)C	424.53	5	2	3.45	0	68.15	0.55	Glycyrrhizae Radix et Rhizoma
Licocoumarone	503731	C20H20O5	CC(=CCC1=C(C2=C(C=C1O)OC(=C2)C3=C(C=C(C=C3)O)O)OC)C	340.37	5	3	2.01	0	83.06	0.55	Glycyrrhizae Radix et Rhizoma
Biochanin A	5280373	C16H12O5	COC1=CC=C(C=C1)C2=COC3=CC(=CC(=C3C2=O)O)O	284.26	5	2	0.77	0	79.9	0.55	Glycyrrhizae Radix et Rhizoma

Formononetin	5280378	C16H12O4	COC1=CC=C(C=C1)C2=COC3=C(C2=O)C=CC(=C3)O	268.26	4	1	1.33	0	59.67	0.55	Glycyrrhizae Radix et Rhizoma
3-Methylkaempferol	5280862	C16H12O6	COC1=C(OC2=CC(=CC(=C2C1=O)O)O)C3=CC=C(C=C3)O	300.26	6	3	0.22	0	100.13	0.55	Glycyrrhizae Radix et Rhizoma
Kaempferol	5280863	C15H10O6	C1=CC(=CC=C1C2=C(C(=O)C3=C(C=C(C=C3O2)O)O)O)O	286.24	6	4	-0.03	0	111.13	0.55	Glycyrrhizae Radix et Rhizoma
Genistein	5280961	C15H10O5	C1=CC(=CC=C1C2=COC3=CC(=CC(=C3C2=O)O)O)O	270.24	5	3	0.52	0	90.9	0.55	Glycyrrhizae Radix et Rhizoma
Genkwanin	5281617	C16H12O5	COC1=CC(=C2C(=C1)OC(=CC2=O)C3=CC=C(C=C3)O)O	284.26	5	2	0.77	0	79.9	0.55	Glycyrrhizae Radix et Rhizoma
Daidzein	5281708	C15H10O4	C1=CC(=CC=C1C2=COC3=C(C2=O)C=CC(=C3)O)O	254.24	4	2	1.08	0	70.67	0.55	Glycyrrhizae Radix et Rhizoma
Licoisoflavone A	5281789	C20H18O6	CC(=CCC1=C(C=CC(=C1O)C2=COC3=CC(=CC(=C3C2=O)O)O)O)C	354.35	6	4	1.09	0	111.13	0.55	Glycyrrhizae Radix et Rhizoma

Luteone	5281797	C20H18O6	CC(=CCC1=C(C2=C(C=C1O)OC=C(C2=O)C3=C(C=C(C=C3)O)O)O)C	354.35	6	4	1.09	0	111.13	0.55	Glycyrrhizae Radix et Rhizoma
Pratensein	5281803	C16H12O6	COC1=C(C=C(C=C1)C2=COC3=CC(=CC(=C3C2=O)O)O)O	300.26	6	3	0.22	0	100.13	0.55	Glycyrrhizae Radix et Rhizoma
Wighteone	5281814	C20H18O5	CC(=CCC1=C(C2=C(C=C1O)OC=C(C2=O)C3=CC=C(C=C3)O)O)C	338.35	5	3	1.64	0	90.9	0.55	Glycyrrhizae Radix et Rhizoma
Kumatakenin B	5282073	C15H10O4	C1=CC(=CC=C1C2=CC(=O)C3=C(O2)C=C(C=C3)O)O	254.24	4	2	1.08	0	70.67	0.55	Glycyrrhizae Radix et Rhizoma
Uralenol	5315126	C20H18O7	CC(=CCC1=C(C(=CC(=C1)C2=C(C(=O)C3=C(C=C(C=C3O2)O)O)O)O)O)C	370.35	7	5	0.56	0	131.36	0.55	Glycyrrhizae Radix et Rhizoma
Lupiwighteone	5317480	C20H18O5	CC(=CCC1=C2C(=C(C=C1O)O)C(=O)C(=CO2)C3=CC=C(C=C3)O)C	338.35	5	3	1.64	0	90.9	0.55	Glycyrrhizae Radix et Rhizoma
Gancaonin F	5317482	C21H16O6	CC1(C=CC2=C(O1)C=C3C(=C2OC)C4=C(C5=C(O4)C=C(C=C5)O)C(=O)O3)C	364.35	6	1	2.29	0	82.04	0.55	Glycyrrhizae Radix et Rhizoma

Glabrone	5317652	C20H16O5	CC1(C=CC2=C(O1)C=CC(=C2O)C3=COC4=C(C3=O)C=CC(=C4)O)C	336.34	5	2	1.64	0	79.9	0.55	Glycyrrhizae Radix et Rhizoma
Glycocoumarin	5317756	C21H20O6	CC(=CCC1=C(C2=C(C=C1O)OC(=O)C(=C2)C3=C(C=C(C=C3)O)O)OC)C	368.38	6	3	2.13	0	100.13	0.55	Glycyrrhizae Radix et Rhizoma
Glycyrrhisoflavone	5317764	C20H18O6	CC(=CCC1=C(C(=CC(=C1)C2=COC3=CC(=CC(=C3C2=O)O)O)O)O)C	354.35	6	4	1.09	0	111.13	0.55	Glycyrrhizae Radix et Rhizoma
Glyzaglabrin	5317777	C16H10O6	C1OC2=C(O1)C(=C(C=C2)C3=COC4=C(C3=O)C=CC(=C4)O)O	298.25	6	2	0.63	0	89.13	0.55	Glycyrrhizae Radix et Rhizoma
Inflacoumarin A	5318437	C20H18O4	CC(=CCC1=CC2=C(C=C1O)OC(=O)C=C2C3=CC=C(C=C3)O)C	322.35	4	2	3.01	0	70.67	0.55	Glycyrrhizae Radix et Rhizoma
Isolicoflavonol	5318585	C20H18O6	CC(=CCC1=C(C=CC(=C1)C2=C(C(=O)C3=C(C=C(C=C3O2)O)O)O)O)C	354.35	6	4	1.09	0	111.13	0.55	Glycyrrhizae Radix et Rhizoma
Kumatakenin	5318869	C17H14O6	COC1=CC(=C2C(=C1)OC(=C(C2=O)OC)C3=CC=C(C=C3)O)O	314.29	6	2	0.47	0	89.13	0.55	Glycyrrhizae Radix et Rhizoma

Licochalcone A	5318998	C21H22O4	CC(C)(C=C)C1=C(C=C(C(=C1)C=CC(=O)C2=CC=C(C=C2)O)OC)O	338.4	4	2	2.92	0	66.76	0.55	Glycyrrhizae Radix et Rhizoma
Licochalcone B	5318999	C16H14O5	COC1=C(C=CC(=C1O)O)C=CC(=O)C2=CC=C(C=C2)O	286.28	5	3	1.27	0	86.99	0.55	Glycyrrhizae Radix et Rhizoma
Licoflavone A	5319000	C20H18O4	CC(=CCC1=CC2=C(C=C1O)OC(=CC2=O)C3=CC=C(C=C3)O)C	322.35	4	2	2.2	0	70.67	0.55	Glycyrrhizae Radix et Rhizoma
Licofuranocoumarin	5319001	C21H20O7	CC(C)(C1CC2=C(O1)C=C3C(=C2OC)C=C(C(=O)O3)C4=C(C=C(C=C4)O)O)O	384.38	7	3	1.4	0	109.36	0.55	Glycyrrhizae Radix et Rhizoma
Licoricone	5319013	C22H22O6	CC(=CCC1=C(C=C(C(=C1OC)C2=COC3=C(C2=O)C=CC(=C3)O)O)OC)C	382.41	6	2	1.53	0	89.13	0.55	Glycyrrhizae Radix et Rhizoma
4'-O-Methylgalbridin	5319664	C21H22O4	CC1(C=CC2=C(O1)C=CC3=C2OCC(C3)C4=C(C=C(C=C4)OC)O)C	338.4	4	1	2.95	0	47.92	0.55	Glycyrrhizae Radix et Rhizoma
3-O-Methylglycyrol	5319666	C22H20O6	CC(=CCC1=C(C=C2C(=C1OC)C3=C(C4=C(O3)C=C(C=C4)O)C(=O)O2)OC)C	380.39	6	1	2.5	0	82.04	0.55	Glycyrrhizae Radix et Rhizoma

Licorisoflavan A	5319704	C27H34O5	CC(=CCC1=C(C=CC(=C1O)C2CC3=C(C(=C(C=C3OC2)OC)CC=C(C)C)OC)O)C	438.56	5	2	3.57	0	68.15	0.55	Glycyrrhizae Radix et Rhizoma
Glycyrol	5320083	C21H18O6	CC(=CCC1=C(C2=C(C=C1O)OC(=O)C3=C2OC4=C3C=CC(=C4)O)OC)C	366.36	6	2	2.55	0	93.04	0.55	Glycyrrhizae Radix et Rhizoma
Licoisoflavone B	5481234	C20H16O6	CC1(C=CC2=C(O1)C=CC(=C2O)C3=COC4=CC(=CC(=C4C3=O)O)O)C	352.34	6	3	1.09	0	100.13	0.55	Glycyrrhizae Radix et Rhizoma
Semilicoisoflavone B	5481948	C20H16O6	CC1(C=CC2=C(O1)C(=CC(=C2)C3=COC4=CC(=CC(=C4C3=O)O)O)O)C	352.34	6	3	1.09	0	100.13	0.55	Glycyrrhizae Radix et Rhizoma
Gancaonin H	5481949	C25H24O6	CC(=CCC1=C(C2=C(C=C1O)OC=C(C2=O)C3=CC4=C(C(=C3)O)OC(C=C4)(C)C)O)C	420.45	6	3	2.09	0	100.13	0.55	Glycyrrhizae Radix et Rhizoma
Licoflavonol	5481964	C20H18O6	CC(=CCC1=C(C2=C(C=C1O)OC(=C(C2=O)O)C3=CC=C(C=C3)O)O)C	354.35	6	4	1.09	0	111.13	0.55	Glycyrrhizae Radix et Rhizoma
Topazolin	5481965	C21H20O6	CC(=CCC1=C(C2=C(C=C1O)OC(=C(C2=O)OC)C3=CC=C(C=C3)O)O)C	368.38	6	3	1.31	0	100.13	0.55	Glycyrrhizae Radix et Rhizoma

2',4',2-Trihydroxychalcone	5811533	C15H12O4	C1=CC=C(C(=C1)C=CC(=O)C2=C(C=C(C=C2)O)O)O	256.25	4	3	1.58	0	77.76	0.55	Glycyrrhizae Radix et Rhizoma
Homobutein	6438092	C16H14O5	COC1=C(C=CC(=C1)C=CC(=O)C2=C(C=C(C=C2)O)O)O	286.28	5	3	1.27	0	86.99	0.55	Glycyrrhizae Radix et Rhizoma
Echinatin	6442675	C16H14O4	COC1=C(C=CC(=C1)O)C=CC(=O)C2=CC=C(C=C2)O	270.28	4	2	1.83	0	66.76	0.55	Glycyrrhizae Radix et Rhizoma
Licochalcone C	9840805	C21H22O4	CC(=CCC1=C(C=CC(=C1OC)C=CC(=O)C2=CC=C(C=C2)O)O)C	338.4	4	2	2.92	0	66.76	0.55	Glycyrrhizae Radix et Rhizoma
Chrysophanol	10208	C15H10O4	CC1=CC2=C(C(=C1)O)C(=O)C3=C(C2=O)C=CC=C3O	254.24	4	2	0.92	0	74.6	0.55	Pinelliae Tuber
Heptadecanoic acid	10465	C17H34O2	CCCCCCCCCCCCCCCCC(=O)O	270.45	2	1	4.44	1	37.3	0.85	Pinelliae Tuber
Eicosanoic acid	10467	C20H40O2	CCCCCCCCCCCCCCCCCCCC(=O)O	312.53	2	1	5.13	1	37.3	0.85	Pinelliae Tuber
Succinic acid	1110	C4H6O4	C(CC(=O)O)C(=O)O	118.09	4	2	-0.54	0	74.6	0.85	Pinelliae Tuber
Octadecane	11635	C18H38	CCCCCCCCCCCCCCCCCC	254.49	0	0	6.92	1	0	0.55	Pinelliae

											Tuber
Ethylpalmitate	12366	C18H36O2	CCCCCCCCCCCCCCCC(=O)OCC	284.48	2	0	4.67	1	26.3	0.55	Pinelliae Tuber
Vinylcyclohexane	12757	C8H14	C=CC1CCCCC1	110.2	0	0	3.67	0	0	0.55	Pinelliae Tuber
2-Methylnonane	13379	C10H22	CCCCCCCC(C)C	142.28	0	0	4.82	1	0	0.55	Pinelliae Tuber
Inosine	135398641	C10H12N4O5	C1=NC2=C(C(=O)N1)N=CN2C3C(C(C(O3)CO)O)O	268.23	7	4	-2.26	0	133.49	0.55	Pinelliae Tuber
6-Methyl-2-heptanone	13572	C8H16O	CC(C)CCCC(=O)C	128.21	1	0	2.07	0	17.07	0.55	Pinelliae Tuber
9-Heptadecanol	136435	C17H36O	CCCCCCCCC(CCCCCCCC)O	256.47	1	1	4.7	1	20.23	0.55	Pinelliae Tuber
Pentadecanoic acid	13849	C15H30O2	CCCCCCCCCCCCCCC(=O)O	242.4	2	1	3.94	0	37.3	0.85	Pinelliae Tuber
Sachaliside 1	14048613	C15H20O7	C1=CC(=CC=C1C=CCOC2C(C(C(C(O2)CO)O)O)O)O	312.32	7	5	-1.05	0	119.61	0.55	Pinelliae Tuber
3-Nonyne	140649	C9H16	CCCCCC#CCC	124.22	0	0	4.38	1	0	0.55	Pinelliae Tuber
Isolariciresinol	160521	C20H24O6	COC1=C(C=C2C(C(C(CC2=C1)CO)CO)C3=CC(=C(C=C3)O)OC)O	360.4	6	4	1.17	0	99.38	0.55	Pinelliae Tuber

Isopulegol	170833	C10H18O	CC1CCC(C(C1)O)C(=C)C	154.25	1	1	2.3	0	20.23	0.55	Pinelliae Tuber
Campesterol	173183	C28H48O	CC(C)C(C)CCC(C)C1CCC2C1(CC C3C2CC=C4C3(CCC(C4)O)C)C	400.68	1	1	6.54	1	20.23	0.55	Pinelliae Tuber
Adenine	190	C5H5N5	C1=NC2=NC=NC(=C2N1)N	135.13	3	2	-1.23	0	80.48	0.55	Pinelliae Tuber
2-Pentylfuran	19602	C9H14O	CCCCCC1=CC=CO1	138.21	1	0	1.84	0	13.14	0.55	Pinelliae Tuber
Farnesane	19773	C15H32	CCC(C)CCCC(C)CCCC(C)C	212.41	0	0	6.19	1	0	0.55	Pinelliae Tuber
Beta-sitosterol	222284	C29H50O	CCC(CCC(C)C1CCC2C1(CCC3C2 CC=C4C3(CCC(C4)O)C)C)C(C)C	414.71	1	1	6.73	1	20.23	0.55	Pinelliae Tuber
Erythritol	222285	C4H10O4	C(C(C(CO)O)O)O	122.12	4	4	-1.91	0	80.92	0.55	Pinelliae Tuber
Stigmasta-5,24-dien-3-ol	22848721	C29H48O	CCC(=C(C)C)CCC(C)C1CCC2C1(CCC3C2CC=C4C3(CCC(C4)O)C)C	412.69	1	1	6.62	1	20.23	0.55	Pinelliae Tuber
2-Methyldecane	23415	C11H24	CCCCCCCCC(C)C	156.31	0	0	5.11	1	0	0.55	Pinelliae Tuber
5-Hydroxymethyl-2-furancarboxaldehy	237332	C6H6O3	C1=C(OC(=C1)C=O)CO	126.11	3	1	-1.06	0	50.44	0.55	Pinelliae Tuber

de											
Benzaldehyde	240	C7H6O	C1=CC=C(C=C1)C=O	106.12	1	0	1.45	0	17.07	0.55	Pinelliae Tuber
Benzene-1,2-diol	289	C6H6O2	C1=CC=C(C(=C1)O)O	110.11	2	2	0.79	0	40.46	0.55	Pinelliae Tuber
Dibutyl phthalate	3026	C16H22O4	CCCCOC(=O)C1=CC=CC=C1C(=O)OCCCC	278.34	4	0	3.43	0	52.6	0.55	Pinelliae Tuber
Choline	305	C5H14NO+	C[N+](C)(C)CCO	104.17	1	1	-3.46	0	20.23	0.55	Pinelliae Tuber
2,6-Di-tert-butyl-4-methylphenol	31404	C15H24O	CC1=CC(=C(C(=C1)C(C)(C)C)O)C(C)(C)C	226.44	0	0	6.44	1	0	0.55	Pinelliae Tuber
Lariciresinol	332427	C20H24O6	COC1=C(C=CC(=C1)CC2COC(C2CO)C3=CC(=C(C=C3)O)OC)O	360.4	6	3	1.17	0	88.38	0.55	Pinelliae Tuber
9,12-Octadecadienoic acid	3931	C18H32O2	CCCCCC=CCC=CCCCCCCCC(=O)O	280.45	2	1	4.47	1	37.3	0.85	Pinelliae Tuber
2,5-Dimethyltetradecane	41836	C16H34	CCCCCCCCCC(C)CCC(C)C	226.44	0	0	6.44	1	0	0.55	Pinelliae Tuber
Pulegone	442495	C10H16O	CC1CCC(=C(C)C)C(=O)C1	152.23	1	0	2.2	0	17.07	0.55	Pinelliae Tuber

Gingerol	442793	C17H26O4	CCCCCC(CC(=O)CCC1=CC(=C(C=C1)O)OC)O	294.39	4	2	2.14	0	66.76	0.55	Pinelliae Tuber
Oleic acid	445639	C18H34O2	CCCCCCCCC=CCCCCCCCC(=O)O	282.46	2	1	4.57	1	37.3	0.85	Pinelliae Tuber
Ferulic acid	445858	C10H10O4	COC1=C(C=CC(=C1)C=CC(=O)O)O	194.18	4	2	1	0	66.76	0.85	Pinelliae Tuber
9-Hexadecenoic acid	4668	C16H30O2	CCCCCCC=CCCCCCCCC(=O)O	254.41	2	1	4.09	0	37.3	0.85	Pinelliae Tuber
Coniferin	5280372	C16H22O8	COC1=C(C=CC(=C1)C=CCO)OC2C(C(C(C(O2)CO)O)O)O	342.34	8	5	-1.32	0	128.84	0.55	Pinelliae Tuber
Linoleic acid	5280450	C18H32O2	CCCCCC=CCC=CCCCCCCCC(=O)O	280.45	2	1	4.47	1	37.3	0.85	Pinelliae Tuber
(E)-p-Coumaryl alcohol	5280535	C9H10O2	C1=CC(=CC=C1C=CCO)O	150.17	2	2	1.44	0	40.46	0.55	Pinelliae Tuber
Octadecanoic acid	5281	C18H36O2	CCCCCCCCCCCCCCCCCC(=O)O	284.48	2	1	4.67	1	37.3	0.85	Pinelliae Tuber
Shogaol	5281794	C17H24O3	CCCCCC=CC(=O)CCC1=CC(=C(C=C1)O)OC	276.37	3	1	2.9	0	46.53	0.55	Pinelliae Tuber
3,4-Dihydroxycinnamyl alcohol	5282096	C9H10O3	C1=CC(=C(C=C1C=CCO)O)O	166.17	3	3	0.83	0	60.69	0.55	Pinelliae Tuber

8-Octadecenoic acid	5282758	C18H34O2	CCCCCCCCCC=CCCCCCCC(=O)O	282.46	2	1	4.57	1	37.3	0.85	Pinelliae Tuber
11-Eicosenoic acid	5282769	C20H38O2	CCCCCCCCC=CCCCCCCCCCC(=O)O	310.51	2	1	5.03	1	37.3	0.85	Pinelliae Tuber
Cis-4-decenal	5362620	C10H18O	CCCCCC=CCCC=O	154.25	1	0	2.59	0	17.07	0.55	Pinelliae Tuber
Dehydrodiconiferyl alcohol	5372367	C20H22O6	COC1=CC(=CC2=C1OC(C2CO)C3=CC(=C(C=C3)O)OC)C=CCO	358.39	6	3	1.09	0	88.38	0.55	Pinelliae Tuber
Stigmasta-5,22-dien-3-ol	53870683	C29H48O	CCC(C=CC(C)C1CCC2C1(CCC3C2CC=C4C3(CCC(C4)O)C)C)C(C)C	412.69	1	1	6.62	1	20.23	0.55	Pinelliae Tuber
7-Hexadecenoic acid	543268	C16H30O2	CCCCCCCCC=CCCCCCC(=O)O	254.41	2	1	4.09	0	37.3	0.85	Pinelliae Tuber
Stigmast-4-en-3-one	5484202	C29H48O	CCC(CCC(C)C1CCC2C1(CCC3C2CCC4=CC(=O)CCC34C)C)C(C)C	412.69	1	0	6.62	1	17.07	0.55	Pinelliae Tuber
1,3,12-Nonadecatriene	548894	C19H34	CCCCCCC=CCCCCCCCC=CC=C	262.47	0	0	5.89	1	0	0.55	Pinelliae Tuber
Daucosterol	5742590	C35H60O6	CCC(CCC(C)C1CCC2C1(CCC3C2CC=C4C3(CCC(C4)OC5C(C(C(C(O5)CO)O)O)O)C)C)C(C)C	576.85	6	4	3.96	1	99.38	0.55	Pinelliae Tuber
Thymidine	5789	C10H14N2O5	CC1=CN(C(=O)NC1=O)C2CC(C(O2)CO)O	242.23	5	3	-1.15	0	104.55	0.55	Pinelliae Tuber

Uridine	6029	C9H12N2O6	C1=CN(C(=O)NC1=O)C2C(C(C(O2)CO)O)O	244.2	6	4	-2.24	0	124.78	0.55	Pinelliae Tuber
Tyrosine	6057	C9H11NO3	C1=CC(=CC=C1CC(C(=O)O)N)O	181.19	4	3	-1.7	0	83.55	0.55	Pinelliae Tuber
Adenosine	60961	C10H13N5O4	C1=NC(=C2C(=N1)N(C=N2)C3C(C(C(O3)CO)O)O)N	267.24	7	4	-2.72	0	139.54	0.55	Pinelliae Tuber
3-Nonanone	61235	C9H18O	CCCCCCC(=O)CC	142.24	1	0	2.39	0	17.07	0.55	Pinelliae Tuber
Phenylalanine	6140	C9H11NO2	C1=CC=C(C=C1)CC(C(=O)O)N	165.19	3	2	-1.11	0	63.32	0.55	Pinelliae Tuber
Cytidine	6175	C9H13N3O5	C1=CN(C(=O)N=C1N)C2C(C(C(O2)CO)O)O	243.22	6	4	-2.29	0	130.83	0.55	Pinelliae Tuber
Bisabolene	62346	C15H24	CC1=CCC(=C(C)CCC=C(C)C)CC1	204.35	0	0	4.53	1	0	0.55	Pinelliae Tuber
Tryptophan	6305	C11H12N2O2	C1=CC=C2C(=C1)C(=CN2)CC(C(=O)O)N	204.23	3	3	-1.66	0	79.11	0.55	Pinelliae Tuber
Hydroxycinnamic acid	637542	C9H8O3	C1=CC(=CC=C1C=CC(=O)O)O	164.16	3	2	1.28	0	57.53	0.85	Pinelliae Tuber
Anethole	637563	C10H12O	CC=CC1=CC=C(C=C1)OC	148.2	1	0	2.67	0	9.23	0.55	Pinelliae Tuber
Citral	638011	C10H16O	CC(=CCCC(=CC=O)C)C	152.23	1	0	2.49	0	17.07	0.55	Pinelliae

											Tuber
Caffeic acid	689043	C9H8O4	C1=CC(=C(C=C1C=CC(=O)O)O)O	180.16	4	3	0.7	0	77.76	0.56	Pinelliae Tuber
N-Acetylglutamate	70914	C7H11NO5	CC(=O)NC(CCC(=O)O)C(=O)O	189.17	5	3	-0.73	0	103.7	0.56	Pinelliae Tuber
10,13-Eicosadienoic acid	72444876	C20H36O2	CCCCCCC=CCC=CCCCCCCCCC(=O)O	308.5	2	1	4.93	1	37.3	0.85	Pinelliae Tuber
Furfural	7362	C5H4O2	C1=COC(=C1)C=O	96.08	2	0	-0.56	0	30.21	0.55	Pinelliae Tuber
Anisic acid	7478	C8H8O3	COC1=CC=C(C=C1)C(=O)O	152.15	3	1	1.32	0	46.53	0.85	Pinelliae Tuber
3-Decyne	75425	C10H18	CCCCCCC#CCC	138.21	1	0	1.84	0	13.14	0.55	Pinelliae Tuber
9-Oxo-nonanoic acid	75704	C9H16O3	C(CCCC=O)CCCC(=O)O	172.22	3	1	1.29	0	54.37	0.85	Pinelliae Tuber
Citronellal	7794	C10H18O	CC(CCC=C(C)C)CC=O	154.25	1	0	2.59	0	17.07	0.55	Pinelliae Tuber
Homogentisic acid	780	C8H8O4	C1=CC(=C(C=C1O)CC(=O)O)O	168.15	4	3	0.47	0	77.76	0.56	Pinelliae Tuber
Benzene-1,4-diol	785	C6H6O2	C1=CC(=CC=C1O)O	110.11	2	2	0.79	0	40.46	0.55	Pinelliae Tuber

1-Octene	8125	C8H16	CCCCCCC=C	112.21	0	0	4.06	0	0	0.55	Pinelliae Tuber
2-Undecanone	8163	C11H22O	CCCCCCCCCC(=O)C	170.29	1	0	2.99	0	17.07	0.55	Pinelliae Tuber
Dodecane	8182	C12H26	CCCCCCCCCCCC	170.33	0	0	5.4	1	0	0.55	Pinelliae Tuber
Docosanoic acid	8215	C22H44O2	CCCCCCCCCCCCCCCCCCCCCC(=O)O	340.58	2	1	5.58	1	37.3	0.85	Pinelliae Tuber
Vanillic acid	8468	C8H8O4	COC1=C(C=CC(=C1)C(=O)O)O	168.15	4	2	0.74	0	66.76	0.85	Pinelliae Tuber
2,6,10-Trimethyltetradecane	85785	C17H36	CCCCC(C)CCCC(C)CCCC(C)C	240.47	0	0	6.68	1	0	0.55	Pinelliae Tuber
Protocatechuic aldehyde	8768	C7H6O3	C1=CC(=C(C=C1C=O)O)O	138.12	3	2	0.18	0	57.53	0.55	Pinelliae Tuber
Aromandendrene	91354	C15H24	CC1CCC2C1C3C(C3(C)C)CCC2=C	204.35	0	0	5.65	1	0	0.55	Pinelliae Tuber
Cycloartenol	92110	C30H50O	CC(CCC=C(C)C)C1CCC2(C1(CCC34C2CCC5C3(C4)CCC(C5(C)C)O)C)C	426.72	1	1	6.92	1	20.23	0.55	Pinelliae Tuber
L-Ephedrine	9294	C10H15N	CC(C(C1=CC=CC=C1)O)NC	165.23	2	2	1.56	0	32.26	0.55	Pinelliae

		O									Tuber
Nicotinamide	936	C6H6N2O	C1=CC(=CN=C1)C(=O)N	122.12	2	1	-0.43	0	55.98	0.55	Pinelliae Tuber
3-Methyleicosane	98417	C21H44	CCCCCCCCCCCCCCCCCC(C)CC	296.57	0	0	7.6	1	0	0.55	Pinelliae Tuber
Palmitic acid	985	C16H32O2	CCCCCCCCCCCCCCCC(=O)O	256.42	2	1	4.19	1	37.3	0.85	Pinelliae Tuber
Pinellic acid	9858729	C18H34O5	CCCCCC(C(C=CC(CCCCCCCC(=O)O)O)O)O	330.46	5	4	2.01	0	97.99	0.56	Pinelliae Tuber
6-Methoxywogonin	11609345	C17H14O6	COC1=C(C(=C2C(=C1O)C(=O)C=C(O2)C3=CC=CC=C3)OC)O	314.29	6	2	0.47	0	89.13	0.55	Scutellariae Radix
Baicalein	5281605	C15H10O5	C1=CC=C(C=C1)C2=CC(=O)C3=C(O2)C=C(C(=C3O)O)O	270.24	5	3	0.52	0	90.9	0.55	Scutellariae Radix
Chrysin	5281607	C15H10O4	C1=CC=C(C=C1)C2=CC(=O)C3=C(C=C(C=C3O2)O)O	254.24	4	2	1.08	0	70.67	0.55	Scutellariae Radix
Scutellarein	5281697	C15H10O6	C1=CC(=CC=C1C2=CC(=O)C3=C(O2)C=C(C(=C3O)O)O)O	286.24	6	4	-0.03	0	111.13	0.55	Scutellariae Radix
Wogonin	5281703	C16H12O5	COC1=C(C=C(C2=C1OC(=CC2=O)C3=CC=CC=C3)O)O	284.26	5	2	0.77	0	79.9	0.55	Scutellariae Radix
Oroxylin A	5320315	C16H12O5	COC1=C(C2=C(C=C1O)OC(=CC2=O)C3=CC=CC=C3)O	284.26	5	2	0.77	0	79.9	0.55	Scutellariae Radix

[7] - Gingerol	11472344	C18H28O4	CCCCCCC(CC(=O)CCC1=CC(=C(C=C1)O)OC)O	308.41	4	2	2.37	0	66.76	0.55	Zingiberis Rhizoma Recens
[6] - Isoshogaol	11694761	C17H24O3	CCCCCC(=O)C=CCCC1=CC(=C(C=C1)O)OC	276.37	3	1	2.9	0	46.53	0.55	Zingiberis Rhizoma Recens
12-Gingerol	118547702	C23H38O4	CCCCCCCCCCCC(CC(=O)CCC1=CC(=C(C=C1)O)OC)O	378.55	4	2	3.49	0	66.76	0.55	Zingiberis Rhizoma Recens
6-Gingerdiol	1369949	C17H15BrN2O2S	CC1=CC(=C2C(=C1)SC(=N2)NC(=O)COC3=CC=C(C=C3)Br)C	391.28	3	1	3.31	0	79.46	0.55	Zingiberis Rhizoma Recens
8-Gingerdione	14440537	C19H28O4	CCCCCCCC(=O)CC(=O)CCC1=CC(=C(C=C1)O)OC	320.42	4	1	2.52	0	63.6	0.55	Zingiberis Rhizoma Recens
10-Gingerdione	14440539	C21H32O4	CCCCCCCCCC(=O)CC(=O)CCC1=CC(=C(C=C1)O)OC	348.48	4	1	2.97	0	63.6	0.55	Zingiberis Rhizoma Recens
1-Dehydro-[10]-gingerdione	14999388	C21H30O4	CCCCCCCCCC(=O)CC(=O)C=CC1=CC(=C(C=C1)O)OC	346.46	4	1	2.89	0	63.6	0.55	Zingiberis Rhizoma Recens

Acetoxy-[10]-gingerol	157009850	C24H38O4	CCCCCCCCCC(CC(=O)CCC1=CC(=C(C=C1)C)OC)OC(=O)C	390.56	4	0	4.16	1	52.6	0.55	Zingiberis Rhizoma Recens
6-Gingerdione	162952	C17H24O4	CCCCCC(=O)CC(=O)CCC1=CC(=C(C=C1)O)OC	292.37	4	1	2.05	0	63.6	0.55	Zingiberis Rhizoma Recens
8-Gingerol	168114	C19H30O4	CCCCCCCC(CC(=O)CCC1=CC(=C(C=C1)O)OC)O	322.44	4	2	2.61	0	66.76	0.55	Zingiberis Rhizoma Recens
10-Gingerol	168115	C21H34O4	CCCCCCCCCC(CC(=O)CCC1=CC(=C(C=C1)O)OC)O	350.49	4	2	3.06	0	66.76	0.55	Zingiberis Rhizoma Recens
8-Paradol	213821	C19H30O3	CCCCCCCCCC(=O)CCC1=CC(=C(C=C1)O)OC	306.44	3	1	3.46	0	46.53	0.55	Zingiberis Rhizoma Recens
1-Dehydro-[6]-gingerdione	22321203	C17H22O4	CCCCCC(=O)C=C(C=CC1=CC(=C(C=C1)O)OC)O	290.35	4	2	1.97	0	66.76	0.85	Zingiberis Rhizoma Recens
1-Dehydro-[8]-gingerdione	44610342	C19H26O4	CCCCCCCC(=O)CC(=O)C=CC1=CC(=C(C=C1)O)OC	318.41	4	1	2.44	0	63.6	0.55	Zingiberis Rhizoma Recens

[4] - Gingerol	46901319	C15H22O4	CCCC(CC(=O)CCC1=CC(=C(C=C1)O)OC)O	266.33	4	2	1.64	0	66.76	0.55	Zingiberis Rhizoma Recens
10-Paradol	51352076	C21H34O3	CCCCCCCCCCCC(=O)CCC1=CC(=C(C=C1)O)OC	334.49	3	1	3.91	0	46.53	0.55	Zingiberis Rhizoma Recens
Diacetoxy-[4]-gingerdiol	5317587	C21H32O6	CCCCCC(CC(CCC1=CC(=C(C=C1)O)OC)OC(=O)C)OC(=O)C	380.48	6	1	2.95	0	82.06	0.55	Zingiberis Rhizoma Recens
[12]-Gingerol	5317599	C23H38O4	CCCCCCCCCCCC(CC(=O)CCC1=CC(=C(C=C1)O)OC)O	378.55	4	2	3.49	0	66.76	0.55	Zingiberis Rhizoma Recens
Methyl diacetoxy-[4]-gingerdiol	5319662	C22H34O6	CCCCCC(CC(CCC1=CC(=C(C=C1)OC)OC)OC(=O)C)OC(=O)C	394.5	6	0	3.17	0	71.06	0.55	Zingiberis Rhizoma Recens
8-Shogaol	6442560	C19H28O3	CCCCCCCC=CC(=O)CCC1=CC(=C(C=C1)O)OC	304.42	3	1	3.37	0	46.53	0.55	Zingiberis Rhizoma Recens
[10] - Shogaol	6442612	C21H32O3	CCCCCCCCCC=CC(=O)CCC1=CC(=C(C=C1)O)OC	332.48	3	1	3.82	0	46.53	0.55	Zingiberis Rhizoma Recens

Methyl [6]-gingerol	71391212	C18H28O4	CCCCCC(CC(=O)CCC1=CC(=C(C=C1)OC)OC)O	308.41	4	1	2.37	0	55.76	0.55	Zingiberis Rhizoma Recens
Acetoxy-[6]-gingerol	91715794	C19H28O5	CCCCCC(CC(=O)CCC1=CC(=C(C=C1)O)OC)OC(=O)C	336.42	5	1	2.5	0	72.83	0.55	Zingiberis Rhizoma Recens
6-Paradol	94378	C17H26O3	CCCCCCCC(=O)CCC1=CC(=C(C=C1)O)OC	278.39	3	1	2.99	0	46.53	0.55	Zingiberis Rhizoma Recens
[4] - Shogaol	9794897	C15H20O3	CCCC=CC(=O)CCC1=CC(=C(C=C1)O)OC	248.32	3	1	2.41	0	46.53	0.55	Zingiberis Rhizoma Recens
12-Shogaol	9975813	C23H36O3	CCCCCCCCCCCC=CC(=O)CCC1=CC(=C(C=C1)O)OC	360.53	3	1	4.25	1	46.53	0.55	Zingiberis Rhizoma Recens
Adenin	190	C5H5N5	C1=NC2=NC=NC(=C2N1)N	135.13	3	2	-1.23	0	80.48	0.55	Zizyphi Fructus
Adenosin	60961	C10H13N5O4	C1=NC(=C2C(=N1)N(C=N2)C3C(C(C(O3)CO)O)O)N	267.24	7	4	-2.72	0	139.54	0.55	Zizyphi Fructus
Ferulic acid	445858	C10H10O4	COC1=C(C=CC(=C1)C=CC(=O)O)O	194.18	4	2	1	0	66.76	0.85	Zizyphi Fructus

P-hydroxycinnamic acid	637542	C9H8O3	C1=CC(=CC=C1C=CC(=O)O)O	164.16	3	2	1.28	0	57.53	0.85	Zizyphi Fructus
Caffeic acid	689043	C9H8O4	C1=CC(=C(C=C1C=CC(=O)O)O)O	180.16	4	3	0.7	0	77.76	0.56	Zizyphi Fructus
Zizyberenalic acid	101936049	C30H44O3	CC(=C)C1CCC2(C1C3CCC4C(C3(CC2)C)(CCC5C4(C(=CC5(C)C)C=O)C)C)C(=O)O	452.67	3	1	5.63	1	54.37	0.85	Zizyphi Fructus
Oleanic acid	10494	C30H48O3	CC1(CCC2(CCC3(C(=CCC4C3(CCC5C4(CCC(C5(C)C)O)C)C)C2C1)C)C(=O)O)C	456.7	3	2	5.82	1	57.53	0.85	Zizyphi Fructus
Urasil	1174	C4H4N2O2	C1=CNC(=O)NC1=O	112.09	2	2	-0.8	0	65.72	0.55	Zizyphi Fructus
4-Hydroxy-3-methoxybenzaldehyde	1183	C8H8O3	COC1=C(C=CC(=C1)C=O)O	152.15	3	1	0.51	0	46.53	0.55	Zizyphi Fructus
Xanthine	1188	C5H4N4O2	C1=NC2=C(N1)C(=O)NC(=O)N2	152.11	3	3	-1.25	0	94.4	0.55	Zizyphi Fructus
Alphitolic acid	12305768	C30H48O4	CC(=C)C1CCC2(C1C3CCC4C(C3(CC2)C)(CCC5C4(CC(C(C5(C)C)O)O)C)C)C(=O)O	472.7	4	3	4.97	1	77.76	0.56	Zizyphi Fructus

Pomonic acid	12314450	C30H46O4	CC1CCC2(CCC3(C(=CCC4C3(CCC5C4(CCC(=O)C5(C)C)C)C)C2C1(C)O)C)C(=O)O	470.68	4	2	4.87	1	74.6	0.85	Zizyphi Fructus
Terminic acid	12314613	C30H48O6	CC1(CCC2(CCC3(C(=CCC4C3(CC(C5C4(CC(C(C5(C)CO)O)O)C)O)C)C2C1)C)C(=O)O)C	504.7	6	5	3.33	1	118.22	0.56	Zizyphi Fructus
Apigenin	12358401	C15H10O5	C1=CC(=CC=C1C2=CC(=O)C3=C(C=C(C=C3O2)O)O)O	270.24	5	3	0.52	0	90.9	0.55	Zizyphi Fructus
4-Hydroxybenzaldehyde	126	C7H6O2	C1=CC(=CC=C1C=O)O	122.12	2	1	0.79	0	37.3	0.55	Zizyphi Fructus
4-Hydroxybenzoic acid	135	C7H6O3	C1=CC(=CC=C1C(=O)O)O	138.12	3	2	0.99	0	57.53	0.85	Zizyphi Fructus
Ursonic acid	13893948	C30H46O3	CC1CCC2(CCC3(C(=CCC4C3(CCC5C4(CCC(=O)C5(C)C)C)C)C2C1C)C)C(=O)O	454.68	3	1	5.73	1	54.37	0.85	Zizyphi Fructus
Ceanothic acid	161352	C30H46O5	CC(=C)C1CCC2(C1C3CCC4C(C3(CC2)C)(CCC5C4(C(C(C5(C)C)O)C(=O)O)C)C)C(=O)O	486.68	5	3	4.86	1	94.83	0.56	Zizyphi Fructus
Fucose	17106	C6H12O5	CC1C(C(C(C(O1)O)O)O)O	164.16	5	4	-1.94	0	90.15	0.55	Zizyphi Fructus

Mannose	18950	C6H12O6	C(C1C(C(C(C(O1)O)O)O)O)O	180.16	6	5	-2.75	0	110.38	0.55	Zizyphi Fructus
Epiceanothic acid	23631167	C30H46O5	CC(=C)C1CCC2(C1C3CCC4C(C3(CC2)C)(CCC5C4(C(C(C5(C)C)O)C(=O)O)C)C)C(=O)O	486.68	5	3	4.86	1	94.83	0.56	Zizyphi Fructus
Benzoic acid	243	C7H6O2	C1=CC=C(C=C1)C(=O)O	122.12	2	1	1.6	0	37.3	0.85	Zizyphi Fructus
Rhamnose	25310	C6H12O5	CC1C(C(C(C(O1)O)O)O)O	164.16	5	4	-1.94	0	90.15	0.55	Zizyphi Fructus
2-Hydroxybenzoic acid	338	C7H6O3	C1=CC=C(C(=C1)C(=O)O)O	138.12	3	2	0.99	0	57.53	0.85	Zizyphi Fructus
Gallic acid	370	C7H6O5	C1=C(C=C(C(=C1O)O)O)C(=O)O	170.12	5	4	-0.16	0	97.99	0.56	Zizyphi Fructus
Glactruronic acid	439215	C6H10O7	C1(C(C(OC(C1O)O)C(=O)O)O)O	194.14	7	5	-2.89	0	127.45	0.56	Zizyphi Fructus
Cinnamic acid	444539	C9H8O2	C1=CC=C(C=C1)C=CC(=O)O	148.16	2	1	1.9	0	37.3	0.85	Zizyphi Fructus
Quercetin	5280343	C15H10O7	C1=CC(=C(C=C1C2=C(C(=O)C3=C(C=C(C=C3O2)O)O)O)O)O	302.24	7	5	-0.56	0	131.36	0.55	Zizyphi Fructus
Traumatic acid	5283028	C12H20O4	C(CCCCC(=O)O)CCCC=CC(=O)O	228.28	4	2	2.03	0	74.6	0.85	Zizyphi Fructus

Zizyberanalic acid	54582219	C30H46O4	CC(=C)C1CCC2(C1C3CCC4C(C3(CC2)C)(CCC5C4(C(C(C5(C)C)O)C=O)C)C)C(=O)O	470.68	4	2	4.87	1	74.6	0.85	Zizyphi Fructus
Glucose	5793	C6H12O6	C(C1C(C(C(C(O1)O)O)O)O)O	180.16	6	5	-2.75	0	110.38	0.55	Zizyphi Fructus
Fructose	5984	C6H12O6	C(C(C(C(C(=O)CO)O)O)O)O	180.16	6	5	-2.91	0	118.22	0.55	Zizyphi Fructus
Benthamic acid	601647	C31H50O4	CC1CCC2(CCC3(C(=CCC4C3(CCC5C4(CCC(C5(C)C)O)C)C)C2C1(C)O)C)C(=O)OC	486.73	4	2	5.16	1	66.76	0.55	Zizyphi Fructus
Galactose	6036	C6H12O6	C(C1C(C(C(C(O1)O)O)O)O)O	180.16	6	5	-2.75	0	110.38	0.55	Zizyphi Fructus
Xylose	644160	C5H10O5	C(C(C(C(C=O)O)O)O)O	150.13	5	4	-2.48	0	97.99	0.55	Zizyphi Fructus
Betulininc acid	64971	C30H48O3	CC(=C)C1CCC2(C1C3CCC4C5(CCC(C(C5CCC4(C3(CC2)C)C)(C)C)O)C)C(=O)O	456.7	3	2	5.82	1	57.53	0.85	Zizyphi Fructus
Arabinose	66308	C5H10O5	C(C(C(C(C=O)O)O)O)O	150.13	5	4	-2.48	0	97.99	0.55	Zizyphi Fructus
Ceanthenic acid	71451218	C29H42O4	CC(=C)C1CCC2(C1C3CCC4C(C3(CC2)C(=O)O)(CCC5C4(C=CC5(C)	454.64	4	2	5.4	1	74.6	0.85	Zizyphi Fructus

			C)C)C)C(=O)O								
Protocatechuic acid	72	C7H6O4	C1=CC(=C(C=C1C(=O)O)O)O	154.12	4	3	0.4	0	77.76	0.56	Zizyphi Fructus
Epicatechin	72276	C15H14O6	C1C(C(OC2=CC(=CC(=C21)O)O)C3=CC(=C(C=C3)O)O)O	290.27	6	5	0.24	0	110.38	0.55	Zizyphi Fructus
Magnoflorine	73337	C20H24NO4+	C[N+]1(CCC2=CC(=C(C3=C2C1CC4=C3C(=C(C=C4)OC)O)O)OC)C	342.41	4	2	-1.71	0	58.92	0.55	Zizyphi Fructus
Guanine	764	C5H5N5O	C1=NC2=C(N1)C(=O)N=C(N2)N	151.13	3	3	-1.31	0	100.45	0.55	Zizyphi Fructus
Hypoxanthine	790	C5H4N4O	C1=NC2=C(N1)C(=O)N=CN2	136.11	3	2	-1.17	0	74.43	0.55	Zizyphi Fructus
Ribose	90428	C5H10O5	C(C(C(C(C=O)O)O)O)O	150.13	5	4	-2.48	0	97.99	0.55	Zizyphi Fructus
Catechin	9064	C15H14O6	C1C(C(OC2=CC(=CC(=C21)O)O)C3=CC(=C(C=C3)O)O)O	290.27	6	5	0.24	0	110.38	0.55	Zizyphi Fructus
Glucuronic acid	94715	C6H10O7	C1(C(C(OC(C1O)O)C(=O)O)O)O)O	194.14	7	5	-2.89	0	127.45	0.56	Zizyphi Fructus

* Lipinski violations: Number of violated criteria among the following four properties (Molecular weight ≦ 500 / H-bond acceptors ≦ 10 / H-bond donors ≦ 5 / MlogP ≦ 4.15).

Supplemental Table S2. Physicochemical and Pharmacokinetic Properties of Active Compounds (TCMSP).

Compound	CID			OB	DL	Herb
Quercetin	5280343	C15H10O7	C1=CC(=C(C=C1C2=C(C(=O)C3=C(C=C(C=C3O2)O)O)O)O)O	46.43	0.28	Bupleuri Radix
Isorhamnetin	5281654	C16H12O7	COC1=C(C=CC(=C1)C2=C(C(=O)C3=C(C=C(C=C3O2)O)O)O)O	49.6	0.31	Bupleuri Radix
Kaempferol	5280863	C15H10O6	C1=CC(=CC=C1C2=C(C(=O)C3=C(C=C(C=C3O2)O)O)O)O	41.88	0.24	Bupleuri Radix
Petunidin	73386	C16H13ClO7	COC1=CC(=CC(=C1O)O)C2=[O+]C3=CC(=CC(=C3C=C2O)O)O.[Cl-]	30.05	0.31	Bupleuri Radix
3,5,6,7,3',4',5'-Heptamethox	389001	C22H24O9	COC1=CC(=CC(=C1OC)OC)C2=C(C(=O)C3=C(C(=C(C=C3O2)OC)OC)OC)OC	31.97	0.59	Bupleuri

yflavone						Radix
5,2',4'-Trihydroxy-6,7,5'-trimethoxyflavone	158311	C18H16O8	COC1=C(C=C(C(=C1)C2=CC(=O)C3=C(C(=C(C=C3O2)OC)OC)O)O)O	48.96	0.41	Bupleuri Radix
Longikaurin A	102117144	C20H28O5	CC1(CCCC23C1C(C(C45C2CCC(C4O)C(=C)C5=O)(OC3)O)O)C	47.72	0.53	Bupleuri Radix
Octalupine	201777	C15H25ClN2O2	C1CC2C3CC(CN2C(=O)C1)C4CC(CCN4C3)O.Cl	47.82	0.28	Bupleuri Radix
Sainfuran	185034	C16H14O5	COC1=CC(=C(C=C1)C2=CC3=CC(=C(C=C3O2)OC)O)O	79.91	0.23	Bupleuri Radix
Saikosaponin C_qt	167927	C48H78O17	CC1C(C(C(C(O1)OC2C(OC(C(C2O)O)OC3CCC4(C(C3(C)C)CCC5(C4C=CC67C5(CC(C8(C6CC(CC8)(C)C)CO7)O)C)C)C)COC9C(C(C(C(O9)CO)O)O)O)O)O)O	30.5	0.63	Bupleuri

						Radix
Cubebin	117443	C20H20O6	C1C(C(C(O1)O)CC2=CC3=C(C=C2)OCO3)CC4=CC5=C(C=C4)OCO5	57.13	0.64	Bupleuri Radix
Stigmasterol	5280343	C15H10O7	C1=CC(=C(C=C1C2=C(C(=O)C3=C(C=C(C=C3O2)O)O)O)O)O	43.83	0.76	Bupleuri Radix
Linoleyl acetate	5319042	C20H36O2	CCCCCC=CCC=CCCCCCCCCOC(=O)C	42.1	0.2	Bupleuri Radix
(+)-Anomalin	5319252	C24H26O7	CC=C(C)C(=O)OC1C(C(OC2=C1C3=C(C=C2)C=CC(=O)O3)(C)C)OC(=O)C(=CC)C	46.06	0.66	Bupleuri Radix
Baicalin	64982	C21H18O11	C1=CC=C(C=C1)C2=CC(=O)C3=C(C(=C(C=C3O2)OC4C(C(C(C(O4)C(=O)O)O)O)O)O)O	40.12	0.75	Bupleuri Radi

						x
Frutinone A	441965	C16H8O4	C1=CC=C2C(=C1)C3=C(C(=O)C4=CC=CC=C4O3)C(=O)O2	65.9	0.34	Ginseng Radix
Girinimbin	96943	C18H17NO	CC1=CC2=C(C3=C1OC(C=C3)(C)C)NC4=CC=CC=C42	61.22	0.31	Ginseng Radix
Kaempferol	5280863	C15H10O6	C1=CC(=CC=C1C2=C(C(=O)C3=C(C=C(C=C3O2)O)O)O)O	41.88	0.24	Ginseng Radix
Dianthramine	441562	C14H11NO6	C1=CC(=C(C=C1O)NC2=C(C=CC(=C2)O)C(=O)O)C(=O)O	40.45	0.2	Ginseng Radix
Ginsenoside Rh2	119307	C36H62O8	CC(=CCCC(C)(C1CCC2(C1C(CC3C2(CCC4C3(CCC(C4(C)C)OC5C(C(C(C(O5)CO)O)O)O)C)C)O)C)O)C	36.32	0.56	Ginseng Radix

Diop	33934	$C_{24}H_{38}O_4$	CC(C)CCCCCOC(=O)C1=CC=CC=C1C(=O)OCCCCCC(C)C	43.59	0.39	Ginseng Radix
Gomisin B	5312542	$C_{20}H_{32}O_2$	CCCCCC=CCC=CCC=CCC=CCCCC(=O)O	31.99	0.83	Ginseng Radix
Stigmasterol	5280794	$C_{29}H_{48}O$	CCC(C=CC(C)C1CCC2C1(CCC3C2CC=C4C3(CCC(C4)O)C)C)C(C)C	43.83	0.76	Ginseng Radix
Beta-sitosterol	222284	$C_{29}H_{50}O$	CCC(CCC(C)C1CCC2C1(CCC3C2CC=C4C3(CCC(C4)O)C)C)C(C)C	36.91	0.75	Ginseng Radix

Supplemental Table S3. List of the common genes of XCHT and DCI.

1,017 Common Genes of XCHT and DCI
ABAT, ABCA1, ABCB1, ABCB11, ABCC1, ABCC2, ABCC3, ABCC4, ABCG1, ABCG2, ABCG5, ABCG8, ABI1, ACACA, ACADM, ACAT1, ACE, ACE2, ACHE, ACP5, ACSL4, ACTB, ACTN1, ADA, ADA2, ADAR, ADARB1, ADGRE5, ADH1A, ADH7, ADIPOQ, ADIPOR1, ADK, ADORA1, ADORA2A, ADORA3, ADRB2, ADRB3, AFP, AGPS, AGXT, AHR, AHCY, AICDA, AIF1, AIFM1, AIM2, AKT1, AKT2, AKT3, AKR1A1, AKR1B1, AKR1B10, ALB, ALDOB, ALDH1B1, ALDH5A1, ALKBH1, ALOX12, ALOX15, ALOX15B, ALOX5, AMH, AMPD1, ANK1, ANXA2, ANXA5, ANGPT2, AOX1, APAF1, APEX1, APOA1, APOB, APOBEC1, APOC3, APOE, APOL1, APP, APRT, AR, ARAF, ARG1, ARNT, ARSH, ATF4, ATG5, ATG7, ATP12A, ATP5ME, AURKB, AVP, B2M, BACE1, BAK1, BAX, BCHE, BCKDHA, BCL2, BCL2L1, BCL2L2, BDNF, BECN1, BGLAP, BHLHE40, BID, BIRC5, BOC, BRCA1, BRCA2, BTK, C1QA, C3, C4A, C5, CACNA1C, CACNA1E, CACNA1H, CACNA1I, CADM1, CALCA, CALML3, CALR, CALU, CAMK2A, CANX, CA1, CA2, CA3, CA9, CASP1, CASP3, CASP7, CASP8, CASP9, CAT, CAV1, CBL, CBLB, CCN2, CCNA2, CCNB1, CCND1, CCR5, CD14, CD163, CD19, CD1D, CD2, CD207, CD209, CD274, CD34, CD36, CD38, CD4, CD40, CD44, CD47, CD69, CD79A, CD86, CD8A, CDA, CDC25C, CDH1, CDH2, CDK1, CDK16, CDK2, CDK4, CDK5, CDK6, CDK7, CDK9, CDKN1A, CDKN1B, CDKN2A, CDKN2B, CEBPA, CEP104, CES1, CES2, CETP, CFH, CFTR, CGAS, CHEK1, CHRM2, CHRNA4, CHRNA7, CHRNB2, CHRNB4, CHSY1, CHUK, CIP2A, CLDN1, CLDN5, CLEC4M, CLEC6A, CLEC7A, CLCN5, CLSPN, CNR1, CNR2, COASY, COL11A1, COL1A1, COL2A1, COMT, COQ10A, COQ2, COQ6, COQ7, COQ8B, COQ9, CPE, CREM, CRH, CRP, CRYZ, CSF1R, CSF2, CSNK2A2, CTSA, CTSD, CTSK, CTNNB1,

CTRL, CXCR4, CXCR5, CXCL1, CXCL10, CXCL12, CXCL8, CXCL9, CYBB, CYCS, CYP1A1, CYP1A2, CYP1B1, CYP19A1, CYP2A6, CYP2B6, CYP2C19, CYP2C8, CYP2C9, CYP2D6, CYP2E1, CYP27A1, CYP3A4, CYP51A1, CYP7A1, DAG1, DAGLB, DAO, DAPK1, DBH, DCN, DCT, DCX, DDIT3, DDC, DEGS1, DERA, DGAT1, DGCR8, DHCR24, DHFR, DHODH, DIABLO, DICER1, DKC1, DKK1, DLEU2, DLG4, DNAH5, DNAH9, DNMT1, DPAGT1, DPP4, DPYD, DPYS, DRD1, DRD2, DUOX2, DUSP12, DUSP3, DUSP4, DUT, DYNC1H1, DYRK1A, ECE1, EDN1, EEF2, EGF, EGFR, EHMT1, EIF2AK3, ELANE, ELAVL1, ELP3, EMP2, ENDOG, ENDOV, ENTPD1, ENTPD2, EPHX1, EPHX2, EPCAM, EPO, ERBB2, ERBB3, ERVW-1, ESAM, ESR1, ESR2, ESRRA, ESRRB, F12, F2, F8, FAAH, FABP2, FABP4, FADD, FAS, FASLG, FASN, FBXO32, FBXO7, FCER1G, FCGR3A, FCN1, FCN2, FCN3, FCSK, FDFT1, FDPS, FEN1, FGF1, FGF2, FGF21, FGF23, FGFR1, FNDC5, FNTA, FOS, FOSB, FOXG1, FOXM1, FOXO1, FOXO3, FOXP3, FST, FTH1, FTO, FUCA1, FUT2, FUT8, G6PD, GAA, GALE, GALK1, GALNS, GALT, GAL, GAPDH, GARS1, GATD3, GBA1, GBGT1, GCH1, GCK, GCLN, GCLC, GCG, GDA, GDF15, GDH2, GFER, GFAP, GGA1, GGA3, GGNBP2, GH1, GH2, GHRL, GIP, GJA1, GJA4, GJA5, GJA8, GJB1, GJB2, GJC1, GJD2, GLCE, GLI1, GLI2, GLP1R, GLRX, GMDS, GOLPH3, GPBAR1, GPER1, GPNMB, GPT, GPX4, GRB2, GRHPR, GRIN1, GRIN2A, GRIN2B, GRIN2D, GSDMD, GSK3B, GSR, GSTM3, GTF2H1, GTF2H2, GUCY1A1, H2AX, HADH, HAVCR2, HBB, HBD, HBMS, HBE1, HCAR2, HDAC1, HDAC9, HELB, HEXA, HIF1A, HMBS, HMGCR, HMGA2, HMGB1, HMOX1, HP, HPD, HPGDS, HPRT1, HPX, HCRTR1, HSD11B1, HSD11B2, HSP90AA1, HSP90AB1, HSP90B1, HSPA5, HSPB1, HPSE, HTR1A, HTR3A, HTT, HYCC1, IAPP, ICAM1, ICMT, IDE, IFNG, IFNA1, IFNLR1, IGF1, IGF1R, IL10, IL17A, IL18, IL1B, IL2, IL22, IL23A, IL2RA, IL3, IL4, IL5, IL6, IL6R, IL13, INS, INSR, IRF1, IRF3, IRS1, ITGA2, ITGAM, ITGB3, ITPR3, JAK2, JUN, KAT5, KAT8, KCNH2, KCNA3, KDR, KDM1A, KEAP1, KIF11, KIT, KLK3, KMO, KNG1,

LARGE1, LBP, LCAT, LCN2, LCT, LDHA, LDLR, LEP, LGALS1, LGALS3, LGALS4, LGALS7, LGALS9, LIG1, LIG4, LIPE, LIPC, LMAN1, LONP1, LPA, LPAR3, LPL, LPO, LRAT, LRP12, LTB4R, LTBP2, LTBP3, LTK, LY96, LYST, LYZ, MAD2L1, MAN1B1, MAN2B1, MAN2C1, MAOA, MAOB, MAP1LC3A, MAP2, MAP2K1, MAP2K4, MAP2K7, MAP3K11, MAP3K14, MAPK1, MAPK3, MAPK8, MAPK9, MAPK10, MAPK11, MAPK12, MAPK13, MAPK14, MARK2, MASP1, MASP2, MBD4, MC1R, MCL1, MCOLN1, MDM2, MDM4, MECR, MET, METAP2, METTL14, METTL3, MFN2, MGLL, MGMT, MIOX, MIR375, MIR4425, MIR638, MIR7-3HG, MIR96, MITF, MKI67, MLXIPL, MME, MOCOS, MOCS1, MOGS, MPC1, MPI, MPO, MRC1, MRC2, MS4A1, MSH2, MSTN, MT1A, MT2A, MT-CYB, MTHFR, MTOR, MTTP, MT-TF, MT-TH, MT-TK, MT-TP, MT-TV, MUTYH, MYB, MYBPC3, MYC, MYD88, MYH6, MYL3, MYLK, MYOD1, NANOS2, NASP, NAT2, NAT8, NCOA1, NCOA4, NCOR2, NDRG1, NES, NEUROD2, NEUROG1, NEUROG2, NFE2L2, NFATC1, NFKB1, NFKB2, NFKBIA, NGF, NKX2-1, NLRC4, NLRP3, NOM1, NOS1, NOS2, NOS3, NOTCH1, NOTCH2, NOX4, NPM1, NQO1, NR1H2, NR1H3, NR1H4, NR1I2, NR3C1, NR3C2, NR5A1, NT5C2, NT5E, NTRK1, NTRK2, NUCB2, ODC1, OFD1, OGA, OGG1, OGT, OMP, OPRM1, ORAI1, OSM, OTC, OXTR, P2RX1, P2RX7, P2RY1, P2RY12, P2RY2, P2RY4, P2RY6, P38, PAH, PARP1, PARP3, PAX6, PCK2, PCNA, PCSK9, PDCD1, PDE10A, PDE1B, PDE2A, PDE4B, PDE4D, PDE5A, PDE6C, PDE9A, PDLIM7, PDP1, PDPK1, PDSS1, PDGFB, PDGFRB, PECAM1, PHB1, PIK3C3, PIK3CA, PIK3CB, PIK3CG, PIK3R1, PINK1, PIWIL1, PKM, PLA2G1B, PLAUR, PLG, PLIN1, PLK2, PLCG1, PML, PMM2, PMP2, PMF1, PNKD, PNLIP, PNO1, PNP, POLB, POLH, POMC, POMGNT2, PON1, PON2, PPARA, PPARD, PPARG, PPARGC1A, PPA1, PPIA, PRDX1, PRDX2, PRF1, PRKAA2, PRKACA, PRKCA, PRKCB, PRKCE, PRKCG, PRKCH, PRKCI, PRKCQ, PRKCZ, PRKG1, PRKN, PRNP, PROKR2, PRSS8, PRTN3, PSMC6, PSMD14, PTCH1, PTEN, PTGER2, PTGS1, PTGS2, PTK2,

PTK2B, PTH, PTPN1, PTPN11, PTPRC, PUS1, PVALB, PVR, PXN, PYCARD, PYGM, QRSL1, RAC2, RAD51, RAD52, RAF1, RALA, RARA, RARG, RAPGEF5, RB1, RBKS, RBMS3, RBL2, REG1A, RELA, REN, RETN, REV1, RHD, RHOA, RMI2, RNGTT, RPA3, RPL13A, RPL4, RPIA, RPSA, RTN2, RUNX2, SART3, SCARB1, SCN9A, SCT, SCD, SDHA, SELE, SELP, SELPLG, SERPINA1, SERPINB1, SERPINC1, SERPIND1, SETD2, SFTPD, SGCB, SGK1, SGTA, SH3GL1, SHBG, SHH, SI, SIK1, SIGLEC12, SIRT1, SIRT2, SIRT3, SKP1, SLC15A1, SLC16A1, SLC16A7, SLC1A2, SLC1A3, SLC22A12, SLC22A2, SLC22A6, SLC22A8, SLC26A5, SLC2A1, SLC2A2, SLC2A3, SLC2A4, SLC29A1, SLC29A2, SLC35A2, SLC35B1, SLC35C1, SLC3A2, SLC45A2, SLC47A1, SLC5A1, SLC5A2, SLC6A1, SLC6A2, SLC6A3, SLC6A4, SLC6A9, SLN, SLCO1A2, SLCO1B1, SLCO1B3, SMAD2, SMAD3, SMAD4, SNCA, SOAT1, SOAT2, SOCS2, SOD1, SOD2, SP1, SP3, SP6, SPTB, SQLE, SQSTM1, SRC, SREBF1, SRY, SST, STAT1, STAT3, STAT6, STING1, ST3GAL4, SUOX, SYK, SYP, TAC1, TADA2A, TANK, TAT, TBK1, TBP, TDG, TERC, TF, TFPI, TFAP4, TG, TGFB1, TGFBR1, TGFBR2, TGIF1, TH, THRB, TIA1, TIMP1, TIMP2, TJP1, TKT, TLR2, TLR4, TLR7, TLR8, TLR9, TMEM37, TNF, TNFSF11, TNFRSF10A, TNFRSF10B, TNFRSF11A, TNFRSF11B, TNNI3, TNNT2, TOP1, TOP2A, TPMT, TPCN2, TP53, TRADD, TRAF6, TREX1, TRPV1, TRPV3, TRPV4, TRPV5, TRP-AGG2-5, TTR, TUBA1A, TUBA1C, TUBA3C, TUBA4A, TUBB, TUBB1, TUBB2A, TUBB2B, TUBB3, TUBB4A, TUBB4B, TUBB6, TUBB8, TWNK, TYMP, TYR, TYRP1, TYMS, UCP1, UGDH, UGT1A, UGT1A1, UGT1A3, UGT1A4, UGT1A6, UGT1A7, UGT1A8, UGT1A9, UGT1A10, UGT2B15, UGT2B17, UGT2B4, UGT2B7, ULK1, UMPS, UNG, UNC119, UXT, VCAM1, VDAC1, VEGFA, VEGFC, VIM, VWF, WEE1, WIF1, WIPI1, WT1, WTAP, XBP1, XDH, XIAP, XYLT1, XYLT2, YAP1, YWHAZ, YTHDF1, YTHDF2, ZFP36, ZMYND8, ZNHIT3

Supplemental Table S4. Top15 GO Biological Process Enrichment of Major Target Genes.

Term	*p*-value	Adjusted *p*-value	Genes
Regulation of miRNA Transcription (GO:1902893)	2.79E-15	3.29E-12	JUN;IL6;STAT3;PPARG;ESR1;TNF;TP53;EGFR
Positive Regulation of Biosynthetic Process (GO:0009891)	2.80E-14	1.62E-11	HSP90AA1;AKT1;ESR1;TNF;SIRT1;TLR4;FOXO1;INS
Positive Regulation of miRNA Transcription (GO:1902895)	4.12E-14	1.62E-11	JUN;IL6;STAT3;PPARG;TNF;TP53;EGFR
Positive Regulation of Transcription by RNA Polymerase II (GO:0045944)	9.70E-14	2.37E-11	JUN;STAT3;ESR1;TNF;SIRT1;EGFR;FOXO1;IL6;AKT1;CTNNB1;PPARG;TP53;TLR4;MAPK3
Positive Regulation of miRNA Metabolic Process (GO:2000630)	1.00E-13	2.37E-11	JUN;IL6;STAT3;PPARG;TNF;TP53;EGFR
Negative Regulation of Apoptotic Process (GO:0043066)	6.74E-13	1.32E-10	IL6;SRC;CAV1;BCL2;AKT1;CTNNB1;TNF;SIRT1;TP53;EGFR;FOXO1
Regulation of Apoptotic Process (GO:0042981)	1.54E-12	2.60E-10	JUN;HSP90AA1;IL6;SRC;BCL2;AKT1;CTNNB1;TNF;SIRT1;TP53;EGFR;FOXO1
Positive Regulation of DNA-templated Transcription (GO:0045893)	3.33E-12	4.91E-10	JUN;STAT3;ESR1;TNF;SIRT1;EGFR;FOXO1;IL6;AKT1;CTNNB1;PPARG;TP53;TLR4;MAPK3

Regulation of Nitric Oxide Biosynthetic Process (GO:0045428)	6.66E-12	8.72E-10	HSP90AA1;CAV1;AKT1;ESR1;TNF;TLR4
Epidermal Growth Factor Receptor Signaling Pathway (GO:0007173)	1.14E-11	1.34E-09	SRC;AKT1;MAPK1;CTNNB1;EGFR;MAPK3
Positive Regulation of Apoptotic Process (GO:0043065)	2.09E-11	2.24E-09	JUN;IL6;BCL2;CTNNB1;PPARG;TNF;SIRT1;TP53;FOXO1
Regulation of Transcription by RNA Polymerase II (GO:0006357)	2.41E-11	2.36E-09	JUN;SRC;CAV1;STAT3;ESR1;TNF;SIRT1;EGFR;FOXO1;IL6;AKT1;CTNNB1;PPARG;TP53;TLR4;MAPK3
ERBB Signaling Pathway (GO:0038127)	3.28E-11	2.97E-09	SRC;AKT1;MAPK1;CTNNB1;EGFR;MAPK3
Positive Regulation of Macromolecule Biosynthetic Process (GO:0010557)	6.42E-11	5.40E-09	IL6;STAT3;AKT1;PPARG;TNF;SIRT1;TP53;TLR4;INS
Negative Regulation of Programmed Cell Death (GO:0043069)	1.23E-10	9.59E-09	IL6;SRC;BCL2;AKT1;CTNNB1;SIRT1;TP53;EGFR;FOXO1

Supplemental Table S5. Top15 GO Cellular Component of Major Target Genes.

Term	*p*-value	Adjusted *p*-value	Genes
Caveola (GO:0005901)	5.87E-07	3.82E-05	SRC;CAV1;MAPK1;MAPK3
Plasma Membrane Raft (GO:0044853)	1.91E-06	5.30E-05	SRC;CAV1;MAPK1;MAPK3
Focal Adhesion (GO:0005925)	2.90E-06	5.30E-05	SRC;CAV1;MAPK1;CTNNB1;EGFR;MAPK3
Cell-Substrate Junction (GO:0030055)	3.26E-06	5.30E-05	SRC;CAV1;MAPK1;CTNNB1;EGFR;MAPK3
Membrane Raft (GO:0045121)	3.35E-05	4.35E-04	SRC;CAV1;TNF;EGFR
Cilium (GO:0005929)	6.35E-05	6.88E-04	AKT1;MAPK1;CTNNB1;EGFR;MAPK3
Intracellular Membrane-Bounded Organelle (GO:0043231)	1.03E-04	8.71E-04	JUN;HSP90AA1;SRC;STAT3;ESR1;SIRT1;EGFR;FOXO1;BCL2;AKT1;MAPK1;CTNNB1;PPARG;TP53;MAPK3
Nucleus (GO:0005634)	1.07E-04	8.71E-04	JUN;HSP90AA1;STAT3;ESR1;SIRT1;EGFR;FOXO1;BCL2;AKT1;MAPK1;CTNNB1;PPARG;TP53;MAPK3
Endoplasmic Reticulum Lumen (GO:0005788)	2.54E-04	0.001837	IL6;MAPK1;INS;MAPK3

Intracellular Organelle Lumen (GO:0070013)	3.52E-04	0.002214	HSP90AA1;IL6;MAPK1;EGFR;INS;MAPK3
Early Endosome (GO:0005769)	3.75E-04	0.002214	CAV1;MAPK1;EGFR;MAPK3
Basolateral Plasma Membrane (GO:0016323)	0.001319	0.007142	CAV1;CTNNB1;EGFR
Late Endosome (GO:0005770)	0.001778	0.00889	SRC;MAPK1;MAPK3
Secretory Granule Lumen (GO:0034774)	0.004816	0.022297	HSP90AA1;MAPK1;INS
Transcription Factor AP-1 Complex (GO:0035976)	0.005488	0.022297	JUN

Supplemental Table S6. Top15 GO Molecular function Enrichment of Major Target Genes.

Term	*p*-value	Adjusted *p*-value	Genes
DNA-binding Transcription Factor Binding (GO:0140297)	5.30E-10	6.63E-08	JUN;STAT3;BCL2;CTNNB1;PPARG;SIRT1;TP53;MAPK3
Ubiquitin Protein Ligase Binding (GO:0031625)	1.03E-08	6.40E-07	JUN;HSP90AA1;BCL2;CTNNB1;TP53;EGFR;FOXO1
Ubiquitin-Like Protein Ligase Binding (GO:0044389)	1.54E-08	6.40E-07	JUN;HSP90AA1;BCL2;CTNNB1;TP53;EGFR;FOXO1
RNA Polymerase II-specific DNA-binding Transcription Factor	1.46E-07	3.61E-06	JUN;STAT3;CTNNB1;PPARG;SIRT1;TP53

Binding (GO:0061629)			
Phosphatase Binding (GO:0019902)	1.46E-07	3.61E-06	STAT3;MAPK1;CTNNB1;EGFR;MAPK3
Protein Phosphatase Binding (GO:0019903)	1.74E-07	3.61E-06	STAT3;CTNNB1;TP53;EGFR;FOXO1
DNA-binding Transcription Activator Activity, RNA Polymerase II-specific (GO:0001228)	9.28E-07	1.66E-05	JUN;STAT3;PPARG;ESR1;TP53;FOXO1
ATPase Binding (GO:0051117)	1.20E-06	1.88E-05	SRC;CAV1;ESR1;EGFR
General Transcription Initiation Factor Binding (GO:0140296)	6.17E-06	8.57E-05	JUN;ESR1;TP53
Protease Binding (GO:0002020)	8.02E-06	1.00E-04	BCL2;TNF;TP53;INS
Kinase Binding (GO:0019900)	9.06E-06	1.03E-04	CAV1;STAT3;AKT1;CTNNB1;ESR1;EGFR
Sequence-Specific Double-Stranded DNA Binding (GO:1990837)	8.71E-05	9.07E-04	JUN;STAT3;PPARG;ESR1;TNF;TP53
MAP Kinase Activity (GO:0004707)	1.04E-04	0.001003	MAPK1;MAPK3
Disordered Domain Specific Binding (GO:0097718)	1.75E-04	0.001561	HSP90AA1;TP53
Transcription Coregulator Binding (GO:0001221)	1.90E-04	0.001585	CTNNB1;PPARG;ESR1

Supplemental Table S7. Top 30 KEGG pathway Enrichment of Major Target Genes.

Term	*p*-value	Adjusted *p*-value	Genes
Proteoglycans in cancer	4.30E-21	7.98E-19	SRC;CAV1;STAT3;ESR1;TNF;EGFR;AKT1;MAPK1;CTNNB1;PRKACA;TP53;TLR4;MAPK3
Lipid and atherosclerosis	8.10E-21	7.98E-19	JUN;HSP90AA1;SRC;STAT3;TNF;IL6;BCL2;AKT1;MAPK1;PPARG;TP53;TLR4;MAPK3
Pathways in cancer	2.71E-19	1.78E-17	JUN;HSP90AA1;STAT3;ESR1;EGFR;FOXO1;IL6;BCL2;AKT1;MAPK1;CTNNB1;PPARG;PRKACA;TP53;MAPK3
Prostate cancer	2.75E-18	1.36E-16	HSP90AA1;BCL2;AKT1;MAPK1;CTNNB1;TP53;EGFR;FOXO1;INS;MAPK3
Hepatitis B	4.56E-18	1.80E-16	JUN;IL6;SRC;STAT3;BCL2;AKT1;MAPK1;TNF;TP53;TLR4;MAPK3
Estrogen signaling pathway	9.82E-17	3.22E-15	JUN;HSP90AA1;SRC;BCL2;AKT1;MAPK1;PRKACA;ESR1;EGFR;MAPK3

Human cytomegalovirus infection	1.81E-16	5.09E-15	IL6;SRC;STAT3;AKT1;MAPK1;CTNNB1;PRKACA;TNF;TP53;EGFR;MAPK3
Chemical carcinogenesis	3.54E-16	8.71E-15	JUN;HSP90AA1;SRC;STAT3;BCL2;AKT1;MAPK1;PRKACA;ESR1;EGFR;MAPK3
Shigellosis	4.88E-16	1.07E-14	JUN;SRC;BCL2;AKT1;MAPK1;TNF;TP53;TLR4;EGFR;FOXO1;MAPK3
AGE-RAGE signaling pathway in diabetic complications	6.37E-16	1.25E-14	JUN;IL6;STAT3;BCL2;AKT1;MAPK1;TNF;FOXO1;MAPK3
HIF-1 signaling pathway	1.42E-15	2.54E-14	IL6;STAT3;BCL2;AKT1;MAPK1;TLR4;EGFR;INS;MAPK3
Thyroid hormone signaling pathway	3.73E-15	6.13E-14	SRC;AKT1;MAPK1;CTNNB1;PRKACA;ESR1;TP53;FOXO1;MAPK3
FoxO signaling pathway	7.77E-15	1.18E-13	IL6;STAT3;AKT1;MAPK1;SIRT1;EGFR;FOXO1;INS;MAPK3
Fluid shear stress and atherosclerosis	1.34E-14	1.89E-13	JUN;HSP90AA1;SRC;CAV1;BCL2;AKT1;CTNNB1;TNF;TP53
Colorectal cancer	2.55E-14	3.35E-13	JUN;BCL2;AKT1;MAPK1;CTNNB1;TP53;EGFR;MAPK3
Salmonella infection	4.23E-14	5.21E-13	JUN;HSP90AA1;IL6;BCL2;AKT1;

			MAPK1;CTNNB1;TNF;TLR4;MAPK3
Kaposi sarcoma-associated herpesvirus infection	2.68E-13	3.11E-12	JUN;IL6;SRC;STAT3;AKT1;MAPK1;CTNNB1;TP53;MAPK3
Focal adhesion	3.88E-13	4.25E-12	JUN;SRC;CAV1;BCL2;AKT1;MAPK1;CTNNB1;EGFR;MAPK3
Prolactin signaling pathway	7.73E-13	8.01E-12	SRC;STAT3;AKT1;MAPK1;ESR1;INS;MAPK3
Yersinia infection	1.16E-12	1.15E-11	JUN;IL6;SRC;AKT1;MAPK1;TNF;TLR4;MAPK3
PI3K-Akt signaling pathway	1.42E-12	1.34E-11	HSP90AA1;IL6;BCL2;AKT1;MAPK1;TP53;TLR4;EGFR;INS;MAPK3
Breast cancer	2.06E-12	1.84E-11	JUN;AKT1;MAPK1;CTNNB1;ESR1;TP53;EGFR;MAPK3
Hepatitis C	3.51E-12	3.01E-11	STAT3;AKT1;MAPK1;CTNNB1;TNF;TP53;EGFR;MAPK3
PD-L1 expression and PD-1 checkpoint pathway in cancer	4.39E-12	3.60E-11	JUN;STAT3;AKT1;MAPK1;TLR4;EGFR;MAPK3
Tuberculosis	1.06E-11	8.10E-11	IL6;SRC;BCL2;AKT1;MAPK1;TNF;TLR4;MAPK3
NOD-like receptor signaling pathway	1.11E-11	8.10E-11	JUN;HSP90AA1;IL6;BCL2;MAPK1;TNF;TLR4;MAPK3

Chagas disease	1.17E-11	8.10E-11	JUN;IL6;AKT1;MAPK1;TNF;TLR4;MAPK3
Longevity regulating pathway	1.17E-11	8.10E-11	AKT1;PPARG;PRKACA;SIRT1;TP53;FOXO1;INS
MAPK signaling pathway	1.19E-11	8.10E-11	JUN;AKT1;MAPK1;PRKACA;TNF;TP53;EGFR;INS;MAPK3
C-type lectin receptor signaling pathway	1.34E-11	8.51E-11	JUN;IL6;SRC;AKT1;MAPK1;TNF;MAPK3
